# Ubiquitin-proteasome system controls ciliogenesis at the initial step of axoneme extension

**DOI:** 10.1038/ncomms6081

**Published:** 2014-10-01

**Authors:** Kousuke Kasahara, Yoshitaka Kawakami, Tohru Kiyono, Shigenobu Yonemura, Yoshifumi Kawamura, Saho Era, Fumio Matsuzaki, Naoki Goshima, Masaki Inagaki

**Affiliations:** 1 Division of Biochemistry, Aichi Cancer Center Research Institute, Nagoya, Aichi 464-8681, Japan; 2 Department of Oncology, Graduate School of Pharmaceutical Sciences, Nagoya City University, Nagoya, Aichi 467-8603, Japan; 3 Molecular Profiling Research Center for Drug Discovery, National Institute of Advanced Industrial Science and Technology, Tokyo 135-0064, Japan; 4 Virology Division, National Cancer Center Research Institute, Tokyo 104-0045, Japan; 5 Electron Microscope Laboratory, RIKEN Center for Developmental Biology, Kobe 650-0047, Japan; 6 Japan Biological Informatics Consortium (JBiC), Tokyo 135-8073, Japan; 7 Laboratory of Cell Asymmetry, RIKEN Center of Developmental Biology, Kobe 650-0047, Japan; 8 Department of Cellular Oncology, Nagoya University Graduate School of Medicine, Nagoya, Aichi 466-8550, Japan

## Abstract

Primary cilia are microtubule-based sensory organelles that organize numerous key signals during developments and tissue homeostasis. Ciliary microtubule doublet, named axoneme, is grown directly from the distal end of mother centrioles through a multistep process upon cell cycle exit; however, the instructive signals that initiate these events are poorly understood. Here we show that ubiquitin-proteasome machinery removes trichoplein, a negative regulator of ciliogenesis, from mother centrioles and thereby causes Aurora-A inactivation, leading to ciliogenesis. Ciliogenesis is blocked if centriolar trichoplein is stabilized by treatment with proteasome inhibitors or by expression of non-ubiquitylatable trichoplein mutant (K50/57R). Started from two-stepped global E3 screening, we have identified KCTD17 as a substrate-adaptor for Cul3-RING E3 ligases (CRL3s) that polyubiquitylates trichoplein. Depletion of KCTD17 specifically arrests ciliogenesis at the initial step of axoneme extension through aberrant trichoplein-Aurora-A activity. Thus, CRL3-KCTD17 targets trichoplein to proteolysis to initiate the axoneme extension during ciliogenesis.

## Abstract (web summary)

Biogenesis of the primary cilium begins after cell cycle exit, but the regulatory steps for its formation are poorly defined. Here the authors show that proteasome-mediated removal of the ciliogenesis inhibitor, trichoplein, from mother centrioles initiates the first step of ciliogenesis.

## Graphical abstract

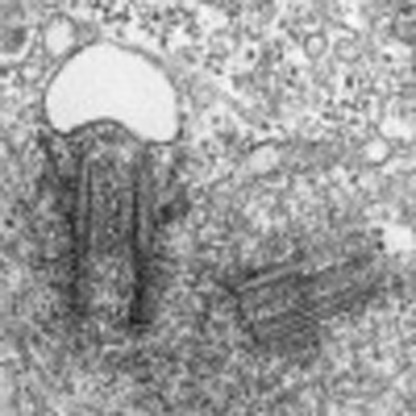

The primary cilium is a membrane-bound, microtubule-based sensory organelle that is composed of nine doublet microtubules, also called ciliary axoneme, elongated directly from the distal end of mother centriole or basal body. Defects in formation, maintenance and function of cilia often results in numerous diseases and developmental disorders, commonly known as ciliopathies^1^,^2^,^3^. Ciliogenesis is evoked upon cell cycle exit and follows a series of ordered steps that have been characterized by detailed ultrastructural analysis of ciliated cells although there are some differences depending on cell type^4^,^5^,^6^. In the intracellular pathway, the recruitment of Golgi-derived ciliary vesicles (CVs) to the distal end of mother centrioles marks the first morphological event during ciliogenesis, followed by the extension of ciliary axoneme and its associated ciliary membrane, and finally, the docking of this complex to the plasma membrane.

Ciliogenesis and cell division are mutually exclusive events as the centrioles must be released from the plasma membrane to function as a mitotic apparatus^7^,^8^,^9^,^10^,^11^. It is therefore conceivable that a set of robust regulatory mechanism is required to suppress the inappropriate ciliogenesis in proliferating cells, and a growing number of centrosomal and ciliary components are actually reported to serve these functions^8^,^12^,^13^,^14^,^15^,^16^,^17^. On the other hand, these proteins must be eliminated when cells exit from cell cycle and form cilia. It has been shown that some protein kinases, such as TTBK2 and MARK4, act to initiate ciliogenesis by excluding CP110 from the mother centrioles^15^,^18^,^19^. Moreover, autophagy-mediated protein degradation was recently reported to remove OFD1 from centriolar satellites to promote ciliogenesis^20^. However, the involvement of ubiquitin-proteasome system (UPS), one of the most important protein degradation system^21^,^22^, appears to be controversial and/or indirect, nevertheless a subset of ubiquitin E3 ligases, including pVHL and MIB-1, has been reported to promote ciliogenesis^23^,^24^,^25^,^26^,^27^.

We have previously shown that trichoplein, originally identified as a keratin-binding protein^28^, is concentrated at the subdistal/medial zone of both mother and daughter centrioles and activates centriolar Aurora-A kinase in growing cells^29^. During ciliogenesis, trichoplein disappears from the mother centrioles, and depletion of this protein induces the aberrant ciliogenesis, whereas overexpression blocks ciliogenesis, indicating that trichoplein negatively regulates ciliogenesis at the mother centrioles.

Trichoplein also controls the recruitment of microtubules to centrioles thorough interaction with Odf2 and ninein in non-ciliated HeLa cells^30^. Other groups have reported that in some tumour cells, trichoplein (also called mitostatin) exists at mitochondria and its overexpression causes the mitochondria fragmentation, thereby inhibiting tumour growth^31^,^32^. A mitochondrial protein VDAC3 is also shown to negatively regulate ciliogenesis at the mother centrioles^17^.

Here we provide definitive evidence that UPS functions to initiate ciliogenesis by removing trichoplein from the mother centrioles. Our global E3 screening has identified KCTD17 as a substrate-adaptor for the Cul3-RING ubiquitin ligases (CRL3s) that polyubiquitylates trichoplein at Lys-50 and Lys-57. The CRL3-KCTD17-meidated trichoplein polyubiquitylation and degradation plays a pivotal role in the initial step of axonemal extension during ciliogenesis through the inactivation of centriolar Aurora-A.

## Results

### UPS targets trichoplein to proteolysis during ciliogenesis

**Figure 1:**
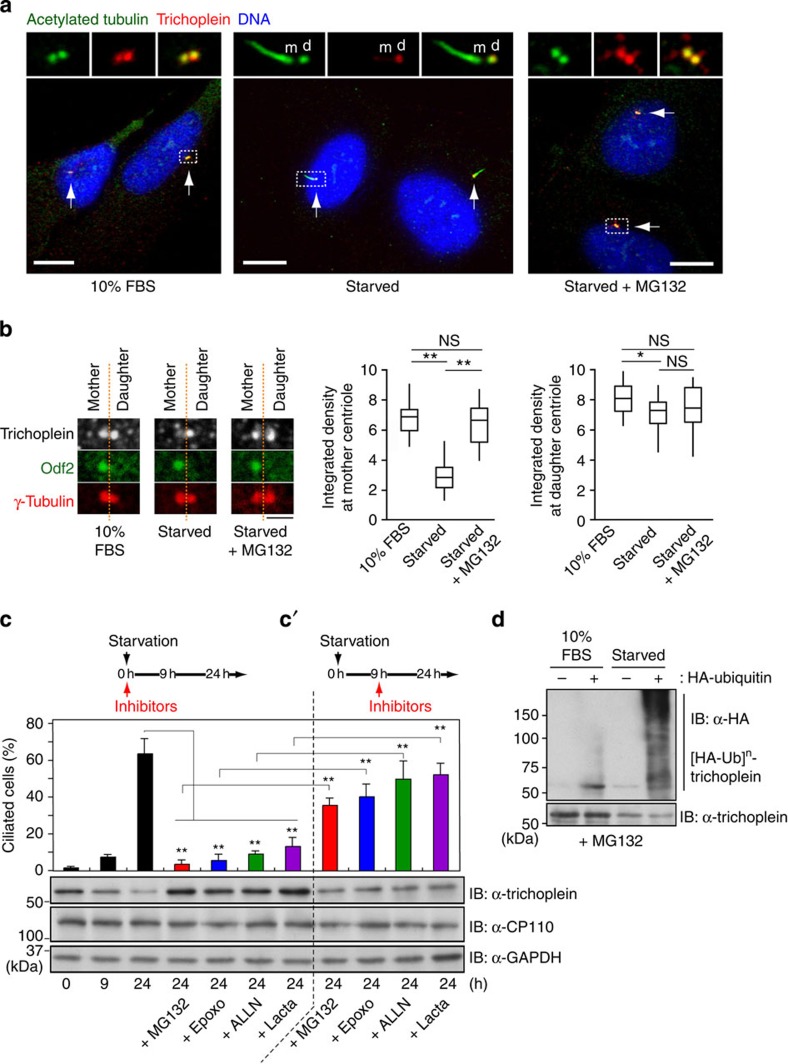
UPS controls ciliogenesis and trichoplein degradation. (**a**–**c**) Effects of proteasome inhibitors (MG132, Epoxomicin (Epoxo), ALLN and Lactacystin (Lacta)) on ciliogenesis and trichoplein levels in RPE1 cells cultured in normal medium (10% fetal bovine serum (FBS)) or subjected to 24 h serum starvation (starved). Respective inhibitors were added at the timing of serum starvation (**a**–**c**) or 9 h after serum starvation (**c′**; see experimental schemes shown in **c** and **c′**). In **a**, representative confocal images of trichoplein (red) with cilia marker acetylated tubulin (green) and DNA (blue) are shown. Arrows indicate the centriolar regions. Mother (m) and daughter (d) centrioles are judged by nucleating cilia in serum-starved condition. In **b**, trichoplein (grey), Odf2 (green) and gamma-tubulin (red) were detected by indirect immunofluorescence and the integrated intensities of trichoplein at mother (judged by presence of Odf2) or daughter centrioles were measured (box-and-whisker plots, *n*=20 from two independent experiments). (**c,c′**) Percentages of ciliated cells (mean±s.e.m. from three or four independent experiments, *n*>200 each) and immunoblotting (IB) analysis of trichoplein, CP110 and glyceraldehyde 3-phosphate dehydrogenase (GAPDH) are shown. (**d**) *In vivo* ubiquitylation assays of endogenous trichoplein in RPE1 cells. Before this assays, cells were cultured in normal medium (10% FBS) or subjected to 6 h serum starvation in the presence of MG132. P**<0.01, 0.01<P*<0.05, NS, not significant, two-tailed unpaired Student’s *t*-tests. Scale bars, 10 μm in **a** and 2 μm in **b**.

When human RPE1 (telomerase reverse transcriptase-immortalized retinal pigment epithelia) cells were exposed to cell cycle signals that induce ciliogenesis by serum starvation^16^,^33^, trichoplein prominently disappeared from the mother centrioles and partly from the daughter centrioles^29^ (mother centriole was judged by the nucleating cilia (Fig. 1a; insets) or the presence of Odf2 (ref. 34; Fig. 1b)). We further found that its protein level was notably decreased (Fig. 1c). However, these reductions were completely blocked in the presence of proteasome inhibitors (MG132, Epoxomicin, ALLN and Lactacystin; Fig. 1a–c). CP110 also disappears from the mother centrioles during ciliogenesis^8^,^15^, but its protein level was not regulated by proteasomal degradation after serum starvation (Fig. 1c). Considering that trichoplein was strikingly polyubiquitylated upon serum starvation (Fig. 1d), the trichoplein removal from mother centriole depends upon the UPS.

### Trichoplein degradation is essential for ciliogenesis

Proteasome inhibition also blocked the ciliogenesis when the respective inhibitors were added at the timing of serum starvation in RPE1 cells (Fig. 1a,c). In contrast, if cells were treated with these inhibitors from the point at which trichoplein was considerably degraded but cilia were not formed yet, for example, 9 h after serum starvation, cilia were newly grown thereafter (Fig. 1c′). We also added these inhibitors 24 h after serum starvation (trichoplein was more degraded at 24 h than at 9 h; see Fig. 1c), and found a marginal effect on ciliogenesis (Supplementary Fig. 1). The inverse correlation between the trichoplein level and ciliogenesis suggests that proteasomal degradation of trichoplein is a critical event for ciliogenesis.

**Figure 2:**
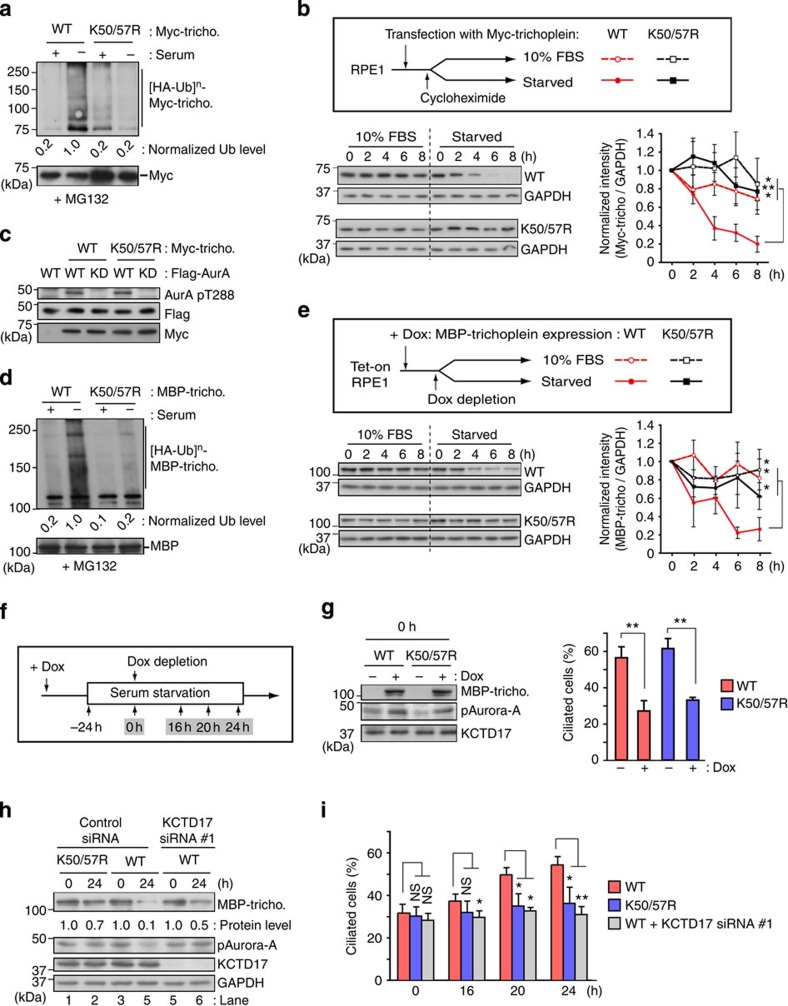
Ubiquitylation-mediated proteolysis of trichoplein is critical for ciliogenesis. (**a**) *In vivo* ubiquitylation assays of myc-trichoplein constructs in RPE1 cells cultured in normal medium (indicated by a plus sign) or subjected to serum starvation (indicated by a minus sign) in the presence of MG132. (**b**) RPE1 cells expressing myc-trichoplein (WT or K50/57R) were treated with cycloheximide in the presence (10% fetal bovine serum (FBS)) or absence of serum (starved) as shown in a scheme. Normalized myc-trichoplein intensities (bottom; mean±s.e.m. in triplicate samples) were evaluated by immunoblotting analysis of myc-trichoplein and glyceraldehyde 3-phosphate dehydrogenase (GAPDH; top). (**c**) Activation of Flag-Aurora-A WT, but not its kinase-dead (KD) mutant, by myc-trichoplein WT and K50/57R in RPE1 cells. Aurora-A activity was judged by auto-phosphorylation at Thr-288 (pAurora-A). (**d**) *In vivo* ubiquitylation assays of MBP-trichoplein-flag constructs in TetOn RPE1 cells cultured in normal medium (indicated by a plus sign) or subjected to serum starvation (indicated by a minus sign) in the presence of MG132. (**e**) Tet-On RPE1 cells expressing MBP-trichoplein-flag (WT or K50/57R) were cultured in doxycycline (Dox)-free culture medium supplemented with (10% FBS) or without serum (starved) as shown in a scheme. Normalized MBP-trichoplein-flag intensities (bottom; mean±s.e.m. in triplicate samples) were evaluated by immunoblotting analysis of MBP-trichoplein-flag and GAPDH (top). (**f**–**i**) Dox-treated Tet-On RPE1 cells were subjected to 24 h serum starvation (0 h), and then cultured in Dox-free serum-starved medium for indicated times as shown in **f**. Immunoblotting analysis shows levels of MBP-trichoplein-flag, pAurora-A, KCTD17 and GAPDH (**g,h**). Graphs show percentages of ciliated cells (mean±s.e.m. from three independent experiment, *n*>200 each). Control or KCTD17 siRNAs were transfected 24 h before serum starvation (**h,i**). P**<0.01, 0.01<P*<0.05, NS, not significant, two-tailed unpaired Student’s *t*-tests.

In addition to these observations, we previously found that overexpression of trichoplein at centrioles blocked the serum starvation-induced ciliogenesis^29^. We therefore investigated whether the decrease in overexpressed trichoplein level permits the ciliogenesis using the non-degradable trichoplein mutant. We generated a series of myc-tagged trichoplein (myc-trichoplein) constructs in which Lys residues were substituted to non-ubiquitylatable Arg, and found that mutation at Lys-50 and Lys-57 (K50/57R) diminished its polyubiquitylation in HEK293T cells (Supplementary Fig. 2a). Polyubiquitylation of myc-trichoplein wild type (WT) was dramatically accelerated by serum starvation in RPE1 cells; however, K50/57R mutation, but not K50R or K57R, diminished this polyubiquitylation (Fig. 2a and Supplementary Fig. 2b). Blockade of new protein synthesis of myc-trichoplein by treatment with cycloheximide rapidly decreased the WT levels in serum-starved but not serum-grown conditions; in contrast, the K50/57R levels were stable in both conditions (Fig. 2b). These results suggest that trichoplein polyubiquitylation at Lys-50 and Lys-57 triggers its degradation. As this mutation had no effect on its ability to activate Aurora-A (Fig. 2c), we decided to use a K50/57R mutant in the following studies. However, we were unable to evaluate the effect on ciliogenesis using cycloheximide because transcriptional control of ciliary gene expression plays a critical role in ciliogenesis^35^,^36^,^37^.

We therefore established the Tet-On RPE1 cell lines that expressed MBP (maltose-binding protein)-tagged trichoplein (MBP-trichoplein) in a doxycycline (Dox)-dependent manner. In these cells, Dox withdrawal promptly decreased the protein level of MBP-trichoplein WT, but not K50/57R, in a polyubiquitylation-dependent manner upon serum starvation (Fig. 2d,e), as was the case with myc-trichoplein in cycloheximide-treated RPE1 cells (Fig. 2a,b), eliminating the concern that the differences in regent (cycloheximide versus Dox) and protein tag affected the trichoplein degradation in RPE1 cells. In the presence of Dox, both overexpressed WT and K50/57R comparably activated Aurora-A and suppressed the serum starvation-induced ciliogenesis (Fig. 2f,g). Twenty-four hours after Dox withdrawal, WT level was drastically decreased to 10% and thereby resulted in Aurora-A inactivation (Fig. 2h; lanes 3 and 4), whereas 70% of K50/57R remained present and kept on activating Aurora-A (Fig. 2h; lanes 1 and 2). Under such conditions, cilia were newly formed only in WT cells (Fig. 2i; compare WT and K50/57R). These results collectively indicate that UPS-mediated proteolysis of trichoplein is essential for ciliogenesis.

### A trichoplein-binding protein KCTD17 controls ciliogenesis

**Figure 3:**
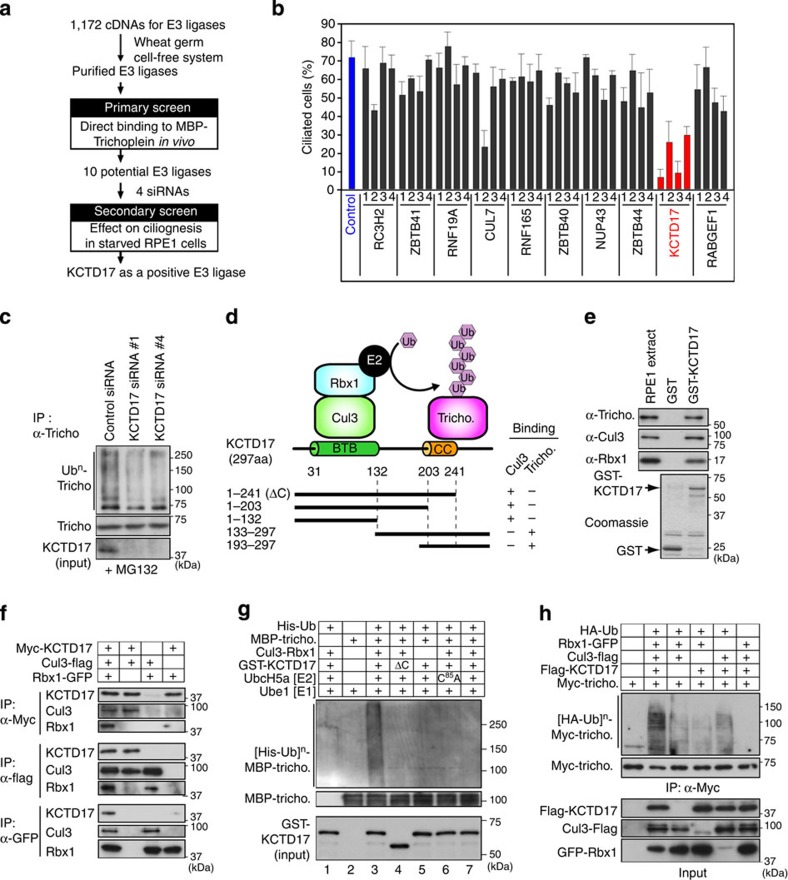
CRL3-KCTD17 polyubiquitylates trichoplein. (**a**) A flowchart of two-stepped global E3 screen. (**b**) RPE1 cells transfected with four distinct siRNAs for indicated genes. 24 h after transfection, cells were subjected to 24 h serum starvation and percentages of ciliated cells were evaluated (mean±s.e.m. from three indicated experiments, *n*>100 each). (**c**) *In vivo* ubiquitylation assays of endogenous trichoplein in control or KCTD17-depleted RPE1 cells subjected to 6 h serum starvation in the presence of MG132. (**d**) Model for CRL3-KCTD17-mediated trichoplein polyubiquitylation (top). Schematic representative of KCTD17 fragments; interactions with Cul3 and trichoplein are shown by a plus sign and a lack of interaction by a minus sign (bottom). (**e**) Pull-down assays of trichoplein, Cul3 and Rbx1 with bacterially purified GST-KCTD17 from RPE1 cell extract. (**f**) Co-immunoprecipitation assays show interactions among Myc-KCTD17, Cul3-Flag and Rbx1-GFP in HEK293T cells. (**g**) *In vitro* ubiquitylation assays of MBP-trichoplein in the presence of indicated proteins. ΔC indicates GST-KCTD17 1–241aa fragment. C^85^A indicates catalytically inactive UbcH5a mutant. (**h**) *In vivo* ubiquitylation assays of Myc-trichoplein in HEK293T cells transfected with indicated cDNA. CC, coiled-coil; IP, immunoprecipitation.

To identify a ubiquitin E3 ligase that controls ciliogenesis through trichoplein degradation, we carried out the two-stepped global E3 screen (Fig. 3a). In the primary screen, 1,172 E3 ligase proteins (including putative E3s; listed in Supplementary Table 1) were purified from the human proteome expression resource library (HuPEX) using wheat germ cell-free expression system^38^,^39^. We evaluated their binding to bacterially purified MBP-trichoplein by protein array, and identified ten potential E3 ligases (Supplementary Fig. 3). We then performed the secondary screen using four distinct small interfering RNAs (siRNAs) per potential E3 ligase; depletion of KCTD17 (K^+^ channel tetramerization domain-containing 17) protein by four independent siRNAs considerably interfered with the serum starvation-induced ciliogenesis both in RPE1 cells and IMR-90 fibroblasts (Fig. 3b and Supplementary Fig. 4). Immunofluoresence analyses of cell cycle markers, including cyclin A and BrdU incorporation, exclude the possibility that the defective ciliogenesis is due to the inappropriate cell cycle re-entry (Supplementary Fig. 5). In addition, KCTD17 silencing attenuated the serum starvation-induced polyubiquitylation of trichoplein (Fig. 3c). Thus, KCTD17 is a novel regulator of ciliogenesis that involves in trichoplein polyubiquitylation.

### CRL3-KCTD17 and UbcH5a/b polyubiquitylate trichoplein

KCTD17 interacted with trichoplein (residues 39–65aa) through its coiled-coil-containing carboxyl-terminal region (residues 193–297aa; Fig. 3d and Supplementary Fig. 6). To examine the mechanism by which KCTD17 modulates polyubiquitylation of trichoplein, we explored the other components of the KCTD17-containing E3 ligase complex. Pull-down and co-immunoprecipitation assays revealed that KCTD17 bound to Cul3, which serves as a scaffold for CRL3s^40^,^41^,^42^,^43^, and that this interaction was mediated via its BTB (broad complex, tramtrack, ‘bric-a-brac’) domain (residues 31–132aa; Fig. 3d,e and Supplementary Fig. 6). KCTD17, Cul3 and a RING protein Rbx1/Roc1 were co-immunoprecipitated with each other (Fig. 3f), indicating that they are in the same complex.

As similar to KCTD17 loss, siRNA-mediated depletions of Cul3 and Rbx1 also interfered with the serum starvation-induced ciliogenesis both in RPE1 and IMR-90 cells (Supplementary Fig. 7), we reasoned that the ternary complex should function as an E3 ligase that polyubiquitylates trichoplein. To test this hypothesis, we performed *in vitro* reconstitution assays using bacterially purified ubiquitin, trichoplein, KCTD17, E1 (Ube1) and respective E2 enzymes, and Cul3-Rbx1 complex purified from HEK293T cells (Fig. 3g and Supplementary Fig. 8). Trichoplein was polyubiquitylated only when all of them were existed in reaction buffer (Fig. 3g; lane 3). KCTD17–trichoplein interaction is essential for the polyubiquitylation because a KCTD17 mutant (ΔC; 1-241aa) that could bind Cul3 but not trichoplein was insufficient (Fig. 3g; lane 4). Among E2s^44^ we tested, UbcH5a and UbcH5b catalyzed *in vitro* polyubiquitylation of trichoplein (Fig. 3g; lane 6 and Supplementary Fig. 8). We further found that trichoplein was most efficiently polyubiquitylated in HEK293T cells when exogenous KCTD17, Cul3 and Rbx1 were co-expressed (Fig. 3h). Taken together, KCTD17 serves as a substrate-adaptor for CRL3 that polyubiquitylates trichoplein (Fig. 3d).

### KCTD17 controls ciliogenesis via trichoplein and Aurora-A

**Figure 4:**
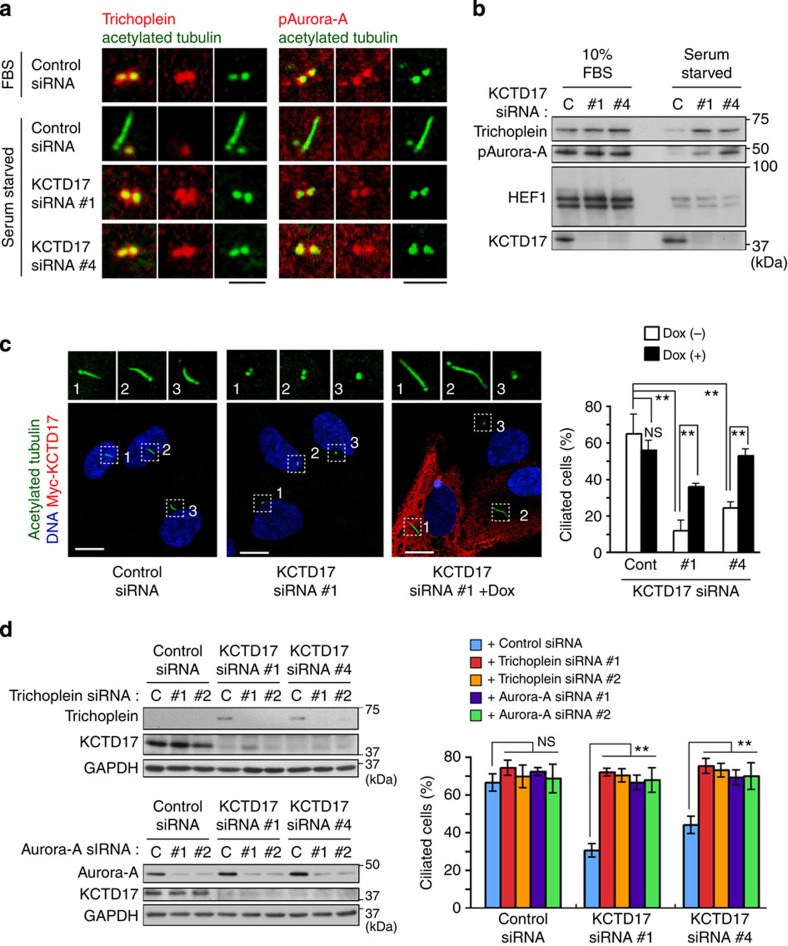
KCTD17 downregulates trichoplein-Aurora-A pathway to promote ciliogenesis. (**a**,**b**) Effects of control or KCTD17 depletion (KCTD17 siRNA #1 or #4) on trichoplein-Aurora-A pathway in RPE1 cells cultured in normal medium (10% fetal bovine serum (FBS)) or subjected to 24 h serum starvation. Representative confocal images of trichoplein or pAurora-A (red) with cilia marker acetylated tubulin (green) are shown in **a**. Immunoblotting analysis of trichoplein, pAurora-A, HEF1 and glyceraldehyde 3-phosphate dehydrogenase (GAPDH) are shown in **b**. (**c**) Dox-dependent expression of myc-KCTD17 reverses the defect of serum starvation-induced ciliogenesis in KCTD17-depleted (#1 and #4) Tet-On RPE1 cells. Left, representative confocal images of acetylated tubulin (green), myc-KCTD17 (red) and DNA (blue) are shown. Right, percentages of ciliated cells (mean±s.e.m. from three independent experiment, *n*>200 each) are shown. (**d**) SiRNA-mediated silencing of Aurora-A (#1 and #2) or trichoplein (#1 and #2) reverses the defect of serum starvation-induced ciliogenesis in KCTD17-depleted (#1 and #4) RPE1 cells. Left, immunoblotting analysis shows levels of Aurora-A, trichoplein, KCTD17 and GAPDH. Right, percentages of ciliated cells (mean±s.e.m. from three independent experiment, *n*>200 each) are shown. P**<0.01, NS, not significant, two-tailed unpaired Student’s *t*-tests. Scale bars indicate 2 μm (**a**) or 10 μm (**c**), respectively.

As noted above, serum starvation caused the trichoplein removal from mother centriole through its proteolysis in control RPE1 cells, but these were prevented in KCTD17-depleted cells (Fig. 4a,b). KCTD17 silencing also disrupted the serum starvation-induced inactivation of centriolar Aurora-A (Fig. 4a,b), as trichoplein is a centriolar activator of Aurora-A^29^. Expression of siRNA-resistant myc-KCTD17 rescued all these phenotypes caused by KCTD17 depletion, namely the defective ciliogenesis (Fig. 4c) and the deregulated trichoplein-Aurora-A pathway (Supplementary Fig. 9). Similar results were also obtained in MBP-trichoplein-expressing Tet-On RPE1 cells (Fig. 2h,i; compare WT and WT+KCTD17 siRNA). HEF-1 is also reported to regulate ciliary dynamics by activating Aurora-A^24^,^33^, but its protein levels were unchanged by KCTD17 depletion (Fig. 4b). These results indicate that KCTD17 contributes to trichoplein degradation and thereby inactivates Aurora-A to induce ciliogenesis.

It has been shown that trichoplein has numerous binding proteins other than Aurora-A^28^,^30^ and its overexpression causes the mitochondrial fragmentation in some tumour cells^31^,^32^ (see also Introduction). We found little changes in their protein levels (Supplementary Fig. 10) and the mitochondrial morphology (Supplementary Fig.11) by overexpression, depletion or stabilization of trichoplein in RPE1 cells under our experimental conditions, suggesting that trichoplein may have the cell type-specific functions. Furthermore, the defective ciliogenesis caused by KCTD17 depletion was completely reversed by co-silencing of trichoplein or Aurora-A (Fig. 4d). Thus, we propose that the KCTD17-trichoplein-Aurora-A pathway controls ciliogenesis in RPE1 cells.

### KCTD17 depletion blocks extension of ciliary axoneme

**Figure 5:**
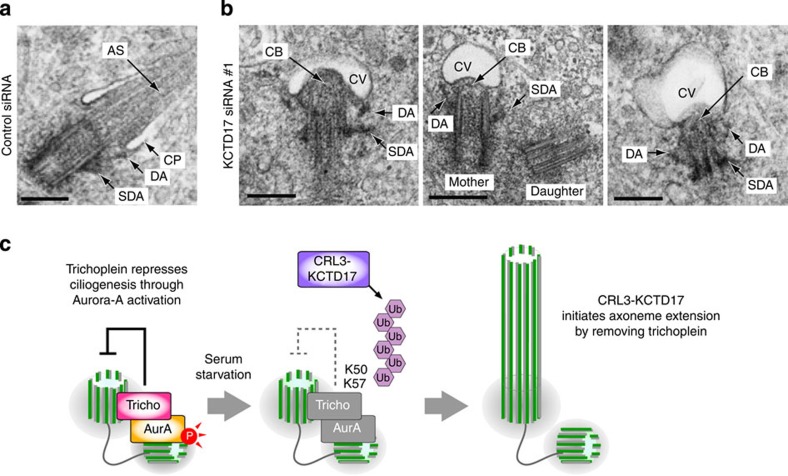
KCTD17 depletion blocks axonemal extension during ciliogenesis. (**a,b**) Transmission electron micrographs of mother centrioles (single sections) in PRE1 cells transfected with control (**a**) or KCTD17 siRNA #1 (**b**), followed by 24 h serum starvation for ciliogenesis. AS, axonemal shaft; CB, ciliary bud; CP, ciliary pocket; CV, ciliary vesicle; DA, distal appendages; SDA, subdistal appendages. Scale bars indicate 500 nm. (**c**) Proposed model.

To get more insight how KCTD17 regulates ciliogenesis, we analyzed mother centrioles and ciliary structures of serum-starved RPE1 cells lacking KCTD17 by transmission electron microscopy. Ciliogenesis follows a series of stereotyped steps that begin with the docking of CVs to the distal appendages of mother centrioles, followed by the formation and extension of ciliary axoneme^5^,^6^ (Supplementary Fig. 12a). After 24 h serum starvation, control cells often demonstrated the mother centrioles that were associated with the ciliary pockets with extended axonemal shafts (Fig. 5a). In contrast, as approximately 90% of KCTD17-depleted cells lacked the elongated ciliary axoneme (see Fig. 3b; KCTD17 siRNA #1), in most of these cells (12 out of 16), the CVs efficiently docked to the mother centrioles, judged by the appearance of appendages, and became invaginated by the accumulated electron-dense materials at the distal end of centrioles (named the ciliary buds; Fig. 5b and Supplementary Fig. 12b). No gross alteration was detected in the ultrastructure of mother centriole. We therefore conclude that KCTD17 is not required for the maturation of mother centriole and the centriole-to-membrane docking, but instead, plays a crucial role in the initial step of axoneme extension during ciliogenesis.

## Discussion

Trichoplein localizes to centrioles and suppresses the aberrant ciliogenesis by activating centriolar Aurora-A in growing cells, but disappears from the mother centrioles when cells are exposed to cell cycle signals that induce ciliogenesis by serum starvation^29^. In this study, we show that the trichoplein removal depends on UPS and that CRL3-KCTD17 targets trichoplein to proteolysis through polyubiquitylation at Lys-50 and Lys-57 during ciliogenesis. If trichoplein is stabilized at mother centrioles by treatment with proteasome inhibitors, expression of non-ubiquitylatable trichoplein mutant (K50/57R) or KCTD17 depletion, in any of these cases, ciliogenesis is blocked. Thus, the trichoplein degradation is an essential event for ciliogenesis. We also demonstrate that Aurora-A silencing completely reverse the defective ciliogenesis caused by trichoplein stabilization, indicating that the KCTD17-trichoplein-Aurora-A cascade controls ciliogenesis at the initial step of axoneme extension (summarized in Fig. 5c).

CP110 is also thought to inhibit the axonemal extension from the mother cenrioles^8^,^15^,^18^,^19^. Similar to trichoplein, CP110 disappears from mother centrioles during ciliogenesis and this removal is essential. However, there are numerous differences between trichoplein and CP110. In contrast to trichoplein localization at the subdistal/medial zone of centrioles^30^, CP110 localizes at the distal end of centrioles and structurally caps the centriole microtubles^14^. Therefore, siRNA-mediated depletion of CP110, but not trichoplein, induces the elongation of centriolar microtubules in non-ciliated cells^12^,^30^,^45^. In addition, the CP110 loss is accompanied by a recruitment of the protein kinase TTBK2 to the distal end of mother centriles^19^, but do not depends on proteasomal degradation (Fig. 1c), during ciliogenesis. Whether or not trichoplein-mediated and CP110-mediated regulations are mutually interrelated in axoneme extension is an interesting area for further experimentation.

CRL3-KCTD17 targets trichoplein to proteolysis in response to serum starvation, but the CRL3-KCTD17 protein levels were unchanged (Supplementary Fig. 13a). Serum starvation had less effect on the myc-KCTD17 localization (Supplementary Fig. 13b) and the trichoplein–KCTD17 interaction (Supplementary Fig. 6b). Thus, serum starvation-induced trichoplein degradation appears to require additional mechanisms. One possibility is that CRL3-KCTD17 activity may be modulated through post-translational modification like phosphorylation by MARK4 or TTBK2 (refs 18, 19). Alternatively, an unidentified deubiquitylating enzyme that counteracts the trichoplein polyubiquitylation may be active in growing cells and become inactive upon serum starvation. It will be future work to investigate whether the trichoplein degradation is regulated by these protein kinases or deubiquitylating enzyme and its physiological contribution to axoneme extension during ciliogenesis.

## Methods

### cDNA

Human cDNAs for KCTD17 (FLJ12242), Cul3 (FLJ76583) and Rbx1 (FLJ96824) were from the Human Gene and Protein Database (HGPD). Full-length KCTD17 was amplified using PCR primers (5′-AAAAGGATCCATGCAGACGCCGCGGCCGGCGATGAGGATGGAGGCCGGGGAG-3′ and 5′-CCGGAATTCTTAGATGGGAACCCCAAGTC-3′) from FLJ12242 as a template. pCGN-HA-Ubiquitin was a kind gift from A. Kikuchi (Osaka University, Japan). Human cDNAs for trichoplein and Aurora-A were previously described^28^,^29^,^30^. Plasmid transfection was performed with Lipofectamine 2000 (Invitrogen) or FuGENE HD (Promega) transfection reagents in HEK293T or RPE1 cell, respectively.

### Cell culture

hTERT-immortalized human retinal pigment epithelial (RPE1) cells were cultured in DMEM and F12 nutrient mix (1:1) supplemented with 10% fetal bovine serum. IMR-90 and HEK293T cells were cultured in DMEM supplemented with 10% fetal bovine serum. MG132 (0.3 μM), Lactacystin (2 μM), ALLN (10 μM) and cycloheximide (200 ng ml^−1^) were purchased from Merck. Epoxomicin (20 nM) was obtained from Peptide Institute Inc.

### Establishment of Tet-On RPE1 cell lines

Tet-On RPE1 cell lines that expressed myc-KCTD17 or MBP-trichoplein-flag (WT or K50/57R) were established with the same procedure described previously^29^,^46^,^47^. The rtTA-advanced segment and the tTS transcriptional silencer segment from pTet-On Advanced and pQC-tTS-IN (BD Clontech) were recombined into the retroviral vector pDEST-PQCXIP and pDEST-PQCXIN, respectively, by the LR reaction (Invitrogen) to generate PQCXIN-Tet-On ADV and PQCXIP-tTS. The Elongation factor 1 alpha promoter (EF) in CSII-EF-MCS (a gift from Hiroyuki Miyoshi, RIKEN BioResource Center, Tsukuba, Japan) was replaced with a Tet-responsive promoter (TRE-Tight) from pTRE-Tight (BD Clontech) followed by a modified RfA fragment (Invitrogen) to make a Tet-responsive lentivirus vector, CSII-TRE-Tight-RfA. Fusion cDNAs with siRNA-resistant KCTD17 and trichoplein were recombined into the lentiviral vector by the LR reaction (Invitrogen) to generate CSII-TRE-Tight-myc-KCTD17 and CSII-TRE-Tight-MBP-trichoplein-3xFLAG, respectively. For induction of myc-KCTD17 or MBP-trichoplein-flag, Tet-On RPE1 cells were treated with 30 or 100 ng ml^−1^ doxycycline (Sigma-Aldrich), respectively.

### Protein purification

Cul3-3xFlag and Rbx1-GFP were coexpressed in HEK293T cells using Lipofectamine 2000. One day after transfection, cells were lysed in cell lysis buffer (20 mM Tris-HCl (pH 7.5), 150 mM NaCl, 2 mM β-glycerophosphate, 50 mM NaF, 1 mM Na_3_VO_4_, 2.5 mM sodium pyrophosphate, 2 mM EDTA, 1 mM EGTA and 1% Triton X-100) containing protease inhibitor cocktail (Nacalai Tesque). Cul3-3xFlag immunocomplexes immobilized on anti-DYKDDDDK-tag antibody beads (Wako Laboratory Chemicals) were washed three times with cell lysis buffer and twice with TBS (50 mM Tris–HCl (pH 7.4) 150 mM NaCl), and then eluted with 100 μg ml^−1^ 3xFlag peptide (Sigma-Aldrich) dissolved in PBS containing 0.1% Tween 20 (PBST). For protein purification from bacteria, GST (glutathione-*S*-transferase)-tagged KCTD17 and MBP-trichoplein^29^ were expressed in DH5α strain (Invitrogen) and BL21 CodonPlus RP stain (Stratagene), respectively. Each protein was purified through the affinity chromatography with glutathione-sepharose 4B (GE Healthcare) or with amylose resin (NewEngland Biolabs).

### Immunoprecipitation and GST pull-down assays

We performed the immunoprecipitation and GST pull-down assays using cell lysis buffer as previously described^46^,^47^. For immunoprecipitation, we used 5 μg of following antibody per assay: anti-GFP (GF090R)-conjugated agarose, c-Myc (MC045) agarose conjugate (Nacalai Tesque), anti-Flag (M2, Sigma-Aldrich) and anti-MBP (1G12, Medical & Biological Laboratories Co. LTD (MBL)).

### *In vitro* ubiquitylation assay

A measure of 12 μg of His-Ubiquitin (LifeSensors Inc), 1 μg of MBP-trichoplein, 0.1 μg of Cul3-3xFlag immunocomplex, 1 μg of GST-KCTD17, 0.4 μg of His-E1 (Ube1; ENZO Life Sciences) and 0.5 μg His-E2 (Ubiquitin-conjugating enzyme sampler pack; Enzo Life Sciences) were incubated in 20 μl of reaction mixture (50 mM Tris–HCl (pH7.5), 5 mM MgCl_2_, 2 mM NaF, 2 mM ATP, 0.6 mM dithiothreitol) at 37 °C for 1 h. The reaction was subjected to immunoprecipitation using anti-trichoplein or anti-MBP before immunoblotting with anti-Ubiquitin.

### *In vivo* ubiquitylation assay

One day after transfection, cells were lysed in the denaturing condition with hot ubiquitin buffer (95 °C) containing 25 mM Tris–HCl (pH 8.0), 1.5% SDS, 0.15% sodium deoxycholate, 0.15% NP-40, 1 mM EDTA, 1 μM okadaic acid and 5 mM *N*-ethylmaleimide. The lysates were diluted (1:10) with cell lysis buffer and used for immunoprecipitation with anti-trichoplein (for endogenous trichoplein), anti-Myc (for myc-trichoplein) and anti-Flag (for MBP-trichoplein-flag). Immunoprecipitates were washed three times with cell lysis buffer containing the denaturing detergent SDS (0.1%), and then analyzed by SDS–polyacrylamide gel electrophoresis. To prevent the proteasomal degradation of polyubiquitylated trichoplein constructs, we treated cells with 10 μM MG132 in the presence or absence of serum for 6 h before cell lysis.

### E3 ubiquitin ligase array

cDNA clones used to screen for E3 ubiquitin ligase to trichoplein were selected from the HuPEX library^39^,^48^. We selected 744 genes to be human E3 ubiquitin ligase by a keyword search of the HGPD ( http://www.HGPD.jp/)^38^, Swiss-Prot database and few papers^49^,^50^,^51^. We have 622 genes to these 744 genes, which were corresponding to 1,172 HuPEX clones containing variant clones (Supplementary Table 1). These HuPEX clones were transferred to the pEW-FG expression vector^39^ harbouring FLAG and GST tags by using the Gateway LR reaction. The protein synthesis was performed by the method of wheat germ protein expression system according to the manufacturer instructions^39^ (CellFree Sciences). Protein array system by using magnetic beads is indicated in Supplementary Fig. 4a. The magnetic beads with glutathione ligand (Promega) were added to reaction mixture containing synthesized protein having FLAG-GST tag, and the synthesized protein was absorbed on the surface of magnetic beads. Beads adsorbed synthesized protein dispensed on the magnet plate (MEIHO Co. Ltd.), which have magnet at the bottom of each well. Each E3 ubiquitin ligase was immobilized at the bottom of magnetic plate via magnetic beads in solution. By using this E3 ubiquitin ligase array, we screened the specific E3 ubiquitin ligase for trichoplein. The array was blocked with PBST and 1% BSA (PBST+BSA). The array was incubated for 1 h at 25 °C with MBP-trichoplein (1.0 mg ml^−1^ in PBST+BSA), then washed in PBST. The array was incubated with HRP-conjugated anti-MBP tag sheep antibody (GE Healthcare; diluted 1:1,000 in PBST+BSA), washed in PBST. Reactive spots were detected with ECL plus (GE Healthcare). The quantity of protein on each spot was assayed by using FLAG M2-Peroxidase (*HRP*)-conjugated monoclonal antibody (Sigma-Aldrich). Reactive spots were normalized by each quantity of protein on each spot.

### Immunoblotting

We performed the immunoblotting as described^46^,^47^. For immunoblotting, we produced a rabbit polyclonal anti-KCTD17 antibody by immunizing bacterially purified KCTD17 (193–297aa) protein (MBL) and a rabbit polyclonal antibody for trichoplein^29^. The list of antibodies with source and conditions of immunoblotting is shown in Supplementary Table 2. In some immunoblotting experiments, we used Can Get Signal immunoreaction enhancer solutions (TOYOBO) for dilution of primary and secondary antibodies. Band intensities were analyzed by densitometry (ImageJ 1.43r, for Macintosh OS X; National Institute of Health, Bethesda, MD, USA). Uncropped versions of the most important blots are shown in Supplementary Figs 14 and 15.

### Microscopy

Immunofluoresence microscopy was performed using confocal microscopy (LSM510 META; Carl Zeiss) equipped with a microscope (Axiovert 200 M; Carl Zeiss), a plan Apochromat × 100/1.4 NA oil immersion lens and LSM image Browser software (Carl Zeiss) as described^29^ with slight modifications. The list of antibodies with source and conditions of indirect immunofluorescence is shown in Supplementary Table 2. In some immunofluorescence experiments, we used Can Get Signal immunostain Solution A (TOYOBO). For induction of primary cilia, RPE1 and IMR-90 cells were seeded on sterile coverslips, grown for 1 day and then subjected to serum starvation. For detection of primary cilia, cells were placed on ice for 20–30 min before fixation with cold methanol, and stained with anti-acetylated tubulin antibody^29^. BrdU incorporation was evaluated using DNA Replication Assay Kit (Millipore). Quantification of fluorescence intensity was performed using ImageJ 1.45r software.

### Transmission electron microscopy

RPE1 cells cultured on coverslips were fixed with 2% fresh formaldehyde and 2.5% glutaraldehyde in 0.1 M sodium cacodylate buffer (pH 7.4) for 2 h at room temperature. After washing with 0.1 M cacodylate buffer (pH 7.4), they were postfixed with ice-cold 1% OsO_4_ in the same buffer for 2 h. The samples were rinsed with distilled water, stained with 0.5% aqueous uranyl acetate for 2 h or overnight at room temperature, dehydrated with ethanol and propylene oxide, and embedded in an embedding kit (Poly/Bed 812; PolySciences, Inc.). After removal of coverslips using ice-cold hydrofluoric acid, ultra-thin sections were cut, doubly stained with uranyl acetate and Reynolds’s lead citrate, and viewed with a transmission electron microscope (JEM-1010; JEOL) with a charge-coupled device camera (BioScan model 792; Gatan, Inc.) at an accelerating voltage of 100 kV.

### SiRNAs

Transfection of siRNA duplexes (final concentration, 20 nM) was performed with Lipofectamine RNAiMAX reagent according to the reverse transfection protocol (Invitrogen). All siRNA duplexes were purchased from Qiagen. Target sequences are listed in Supplementary Table 3.

## Author contributions

M.I. conceived the project. K.K. and M.I. interpreted data and wrote the manuscript. K.K. performed much of the experiments. K.K., T.K, S.E. and F.M. provided experimental materials. Y.-t.K., Y.-f.K. and N.G. conducted protein array screen. S.Y. executed TEM analysis.

## Additional information

**How to cite this article:** Kasahara, K. *et al.* Ubiquitin-proteasome system controls ciliogenesis at the initial step of axoneme extension. *Nat. Commun.* 5:5081 doi: 10.1038/ncomms6081 (2014).

## Supplementary Material

Supplementary Information Supplementary Figures 1-15, Supplementary Tables 1-3 and Supplementary References
