# Supplementary material for: Ubiquitin-proteasome system controls ciliogenesis at the initial step of axoneme extension
Source: Nat Commun. 2014 Oct 1;5:5081. doi: 10.1038/ncomms6081 (PMC4205846; doi:10.1038/ncomms6081)
Supplement: Supplementary Information — Supplementary Figures 1-15, Supplementary Tables 1-3 and Supplementary References [file ncomms6081-s1.pdf]

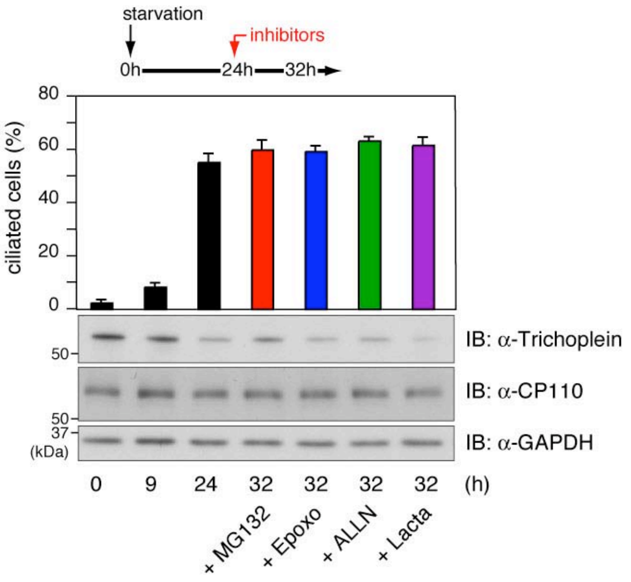

Supplementary Figure 1 | Effect of proteasome inhibitors on ciliogenesis. After 24 h serum starvation, RPE1 cells were treated with proteasome inhibitors (MG132, Epoxomicin [Epoxo], ALLN and Lactacystin [Lacta]) for 8 h as shown in the experimental scheme. Percentages of ciliated cells (mean ± s.e.m. from three independent experiments, n > 200 each) and immunoblotting analysis of trichoplein, CP110 and GAPDH are shown.

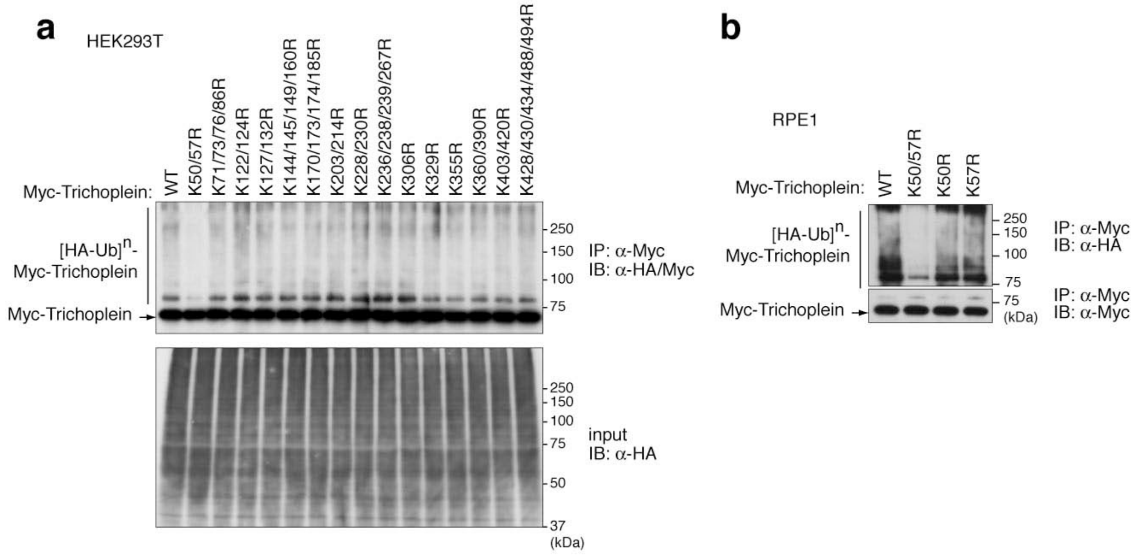

Supplementary Figure 2 | K50/57R mutation attenuated trichoplein polyubiquitylation. (**a**) Wild-type myc-trichoplein (WT) or its mutants were co-expressed with HA-ubiquitin in HEK293T cells. After treatment with MG132 for 6 h, cells were subjected to *in vivo* ubiquitylation assays. (**b**) Myc-trichoplein WT or its mutants were co-expressed with HA-ubiquitin in RPE1 cells. After treatment with MG132 for 6 h in the absence of serum, cells were subjected to *in vivo* ubiquitylation assays.

a

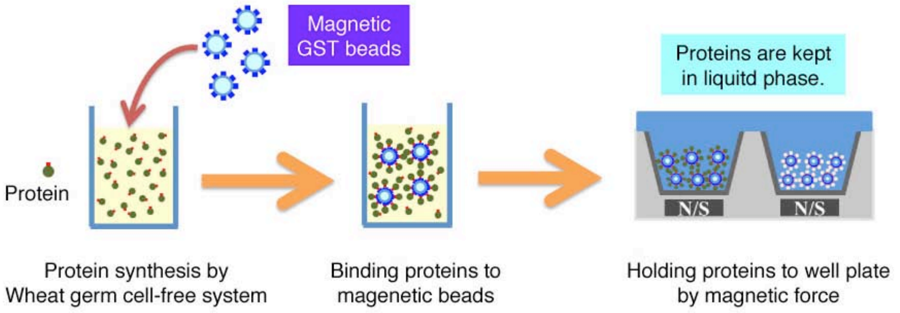

b

MBP-Trichoplein-binding proteins

	gene symbol	FLJ no.	
10 potential E3 ligases	RC3H2	23389AAAN	Ring finger and CCCH-type zinc finger domain 2
	ZBTB41	36199AAAN	Zinc finger and BTB domain containing 41
	RNF19A	23417AAAN	Ring finger protein19A
	CUL7	76468AAAN	Cullin 7
	RNF165	45559AAAN	Ring finger protein 165
	ZTB40	37954AAAN	Zinc finger and BTB domain containing 40
	NUP43	16491AAAN	Nucleoporin 43kDa
	ZBTB44	95555AAAF	Zinc finger and BTB domain containing 44
	KCTD17	12242AAAN	Potassium chanel tetramerization domain containing 17
	RABGEF1	10840AAAN	Rab guanine nucleotide exchange factor (GEF) 1
positive control	AURKA	80023AAAF	Inoko A et al, J Cell BIol, 197: 391-405 (2012)
	KRT18	26076AAAF	Nishizawa M et al, J Cell Sci, 118: 1081-1090 (2005)
	ODF2		Ibi M et al, J Cell Sci, 124: 857-864 (2011)

Supplementary Figure 3 | Screening for E3 ligases that involve in trichoplein polyubiquitylation and ciliogenesis. (**a**) Experimental scheme of protein array; see "Methods" section in details. (**b**) Ten E3 ligases identified as MBP-trichoplein-interacting proteins are shown. This system could detect interactions between MBP-trichoplein and the known trichoplein-interacting protein, such as Aurora-A (AURKA)[1], Keratin-18 (KRT18)[2] and Odf2 (ODF2)[3].

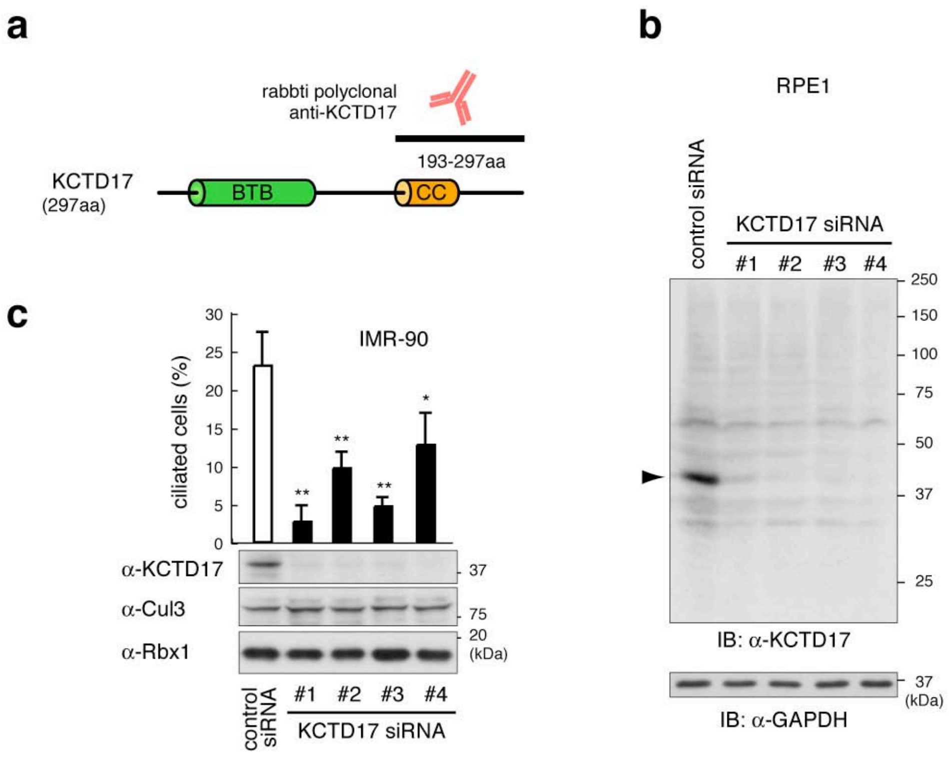

Supplementary Figure 4 | Depletion of KCTD17 protein blocks ciliogenesis. (**a,b**) Bacterially purified KCTD17 fragment (193-297aa) was immunized for production of rabbit polyclonal anti-KCTD17 antibody. This antibody specifically detected KCTD17 in control (control siRNA) but not KCTD17-depleted (KCTD17 siRNA #1-4) RPE1 cells by immunoblotting analysis in (**b**). (**c**) Effect of KCTD17 depletion on ciliogenesis in IMR-90 fibroblasts subjected to 24 h serum starvation. Percentages of ciliated cells (mean ± s.e.m. from three indicated experiments, n > 100 each) are shown in top. p** < 0.01, 0.01 < p* < 0.05, two-tailed unpaired student's *t*-tests.

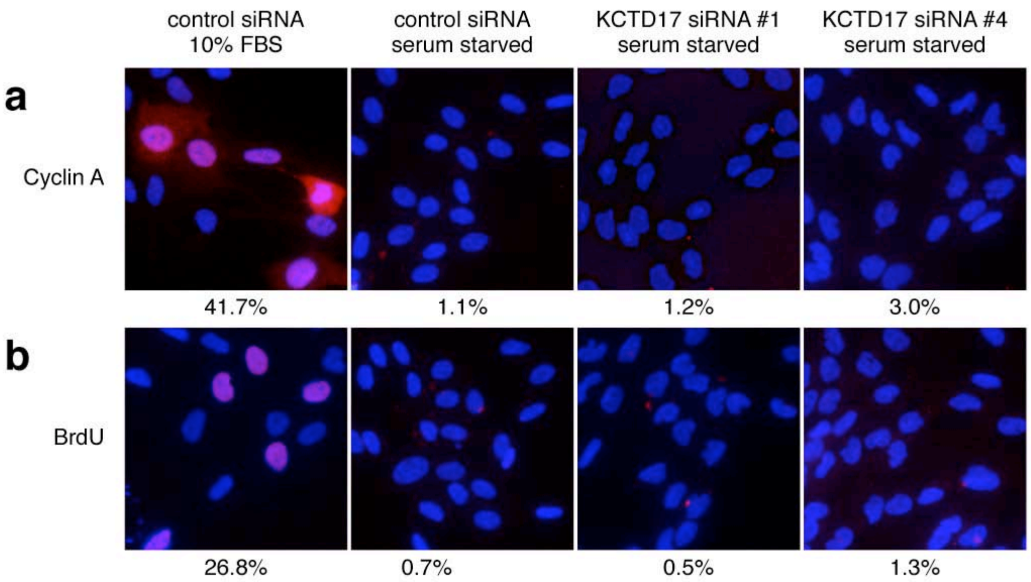

Supplementary Figure 5 | Immunofluorescence analysis of cell cycle markers in KCTD17-depleted cells. RPE1 cells transfected with control or KCTD17 siRNAs (#1 or #4) were cultured for normal medium (10% FBS) or subjected to 24 h serum starvation. Representative confocal images of cyclin A (**a**, red) or incorporated BrdU (**b,** red) with DNA (blue) and percentages of positive cells are shown (mean values from three-independent experiments, n > 200 each).

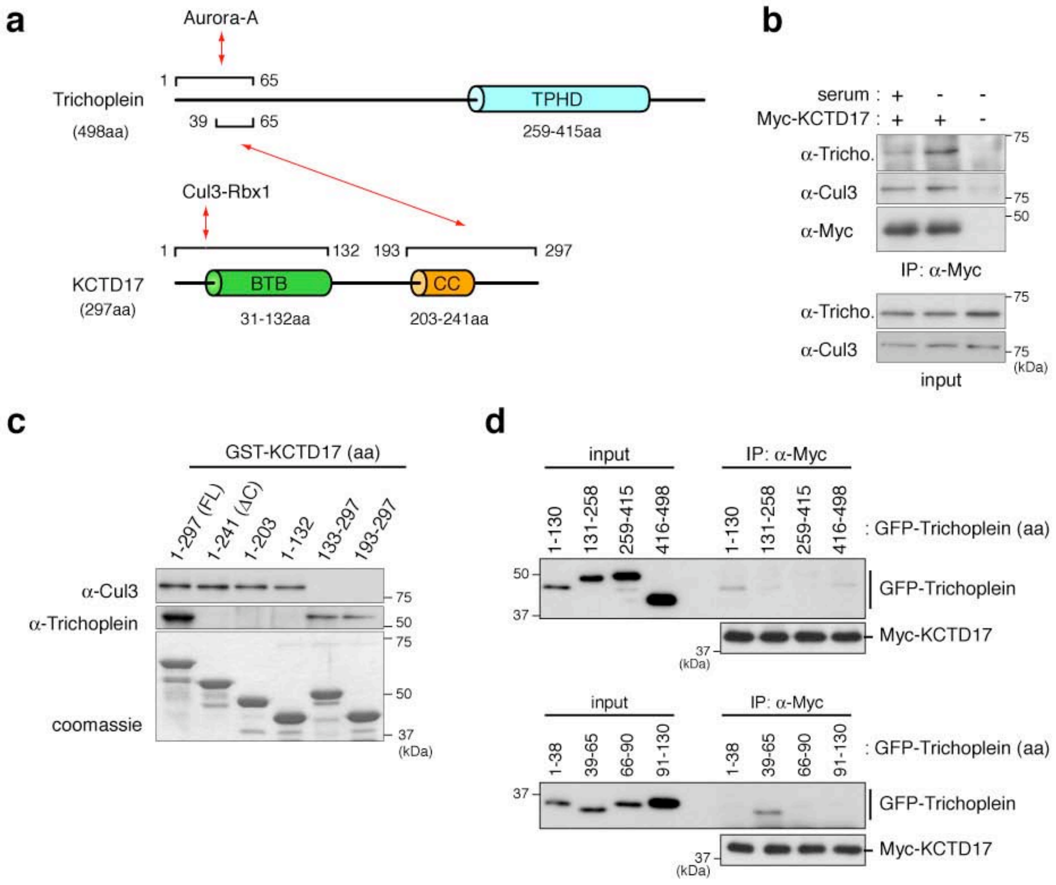

Supplementary Figure 6 | KCTD17 interacts with trichoplein and Cul3. (**a**) A summary of the established interactions of KCTD17, Cul3, trichoplein and Aurora-A. (**b**) Co-immunoprecipitation assays show Myc-KCTD17 interaction with trichoplein and Cul3 in Tet-On RPE1 cells. Myc-KCTD17 expression was induced by Dox treatment (indicated by a plus sign). (**c**) Pull-down assays using bacterially purified GST-tagged KCTD17 truncation mutants with RPE1 cell extract. KCTD17 interactions with Cul3 and trichoplein are shown by immunoblotting analysis. (**d**) Interactions between GFP-trichoplein mutants and Myc-KCTD17 were analyzed by co-immunoprecipitation assays with anti-Myc.

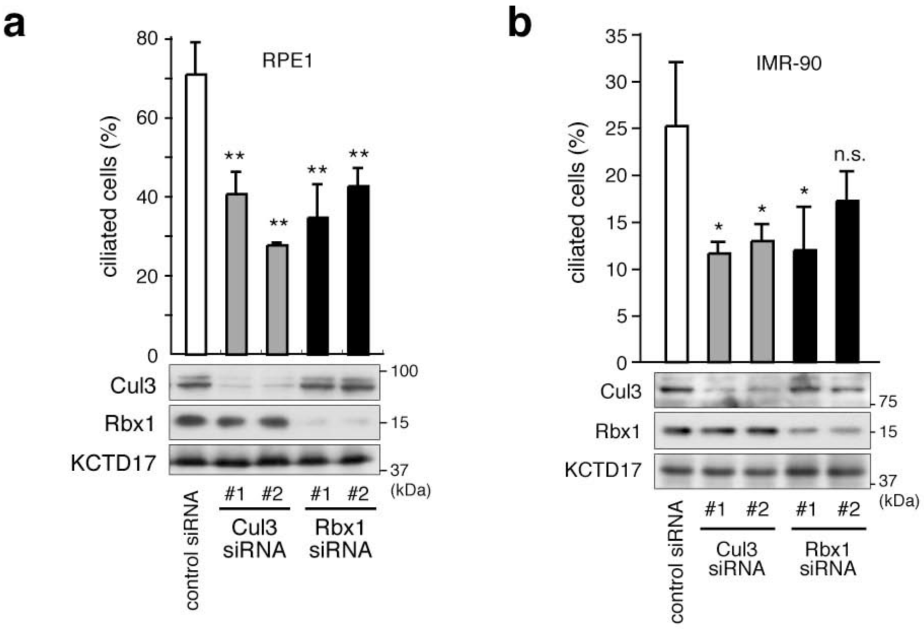

Supplementary Figure 7 | Depletion of Cul3 or Rbx1 prevents ciliogenesis. RPE1 (**a**) and IMR-90 (**b**) cells were transfected with control, Cul3 (#1 or #2), or Rbx1 (#1 or #2) siRNAs, and then subjected to 24 h serum starvation. Graphs show percentages of ciliated cells (mean ± s.e.m. from three indicated experiments, n > 200 each). Immunoblotting analysis show the protein levels of KCTD17, Cul3 and Rbx1. p** < 0.01, 0.01 < p* < 0.05, n.s., not significant, two-tailed unpaired student's *t*-tests.

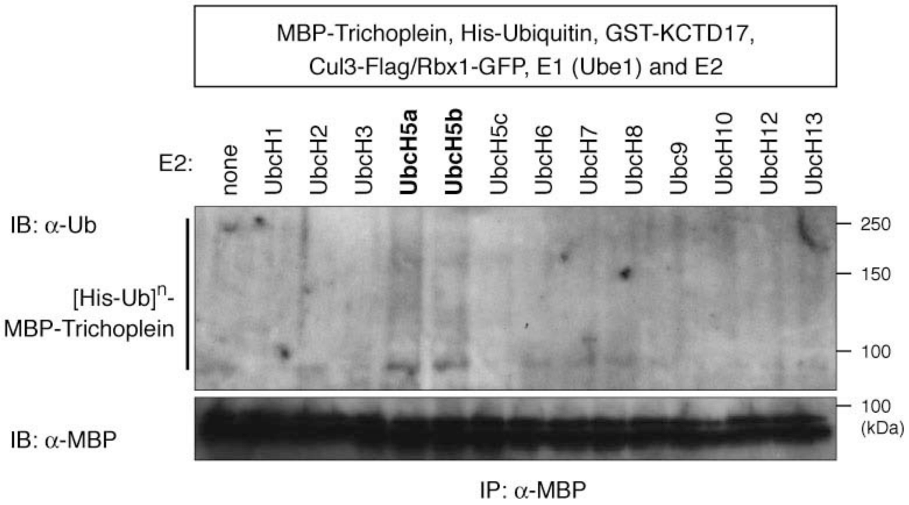

Supplementary Figure 8 | UbcH5a and UbcH5b function as E2 enzymes that polyubiquitylate trichoplein *in vitro*. Recombinant MBP-trichoplein was mixed with His-ubiquitin, GST-KCTD17 and E1 (His-Ube1) purified from *E. coli* and Cul3-3xFlag/Rbx1-GFP complex purified from HEK293T cells in reaction mixture. Mixture was subjected to *in vitro* ubiquitylation assays with indicated E2 (His-tagged). Anti-MBP immunoprecipitates were analyzed by immunoblotting with anti-Ubiquitin and anti-MBP.

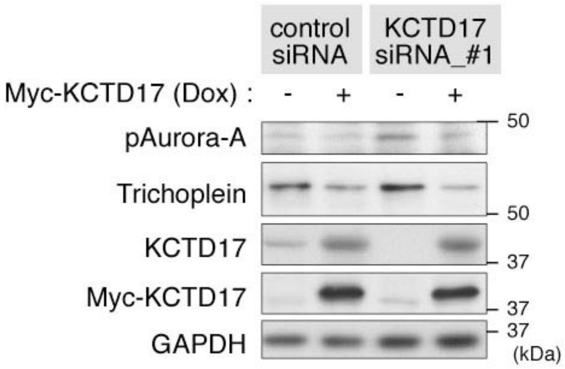

Supplementary Figure 9 | Myc-KCTD17 expression reverses aberrant trichoplein-Aurora-A activity in KCTD17-depleted cells. 24 h after transfection with control or KCTD17 (#1) siRNAs, Tet-On RPE1 cells were subjected to 24 h serum starvation. Myc-KCTD17 expression was induced by Dox addition 8 h after siRNA transfection. Levels of pAurora-A, trichoplein, KCTD17 (endogenous KCTD17 plus myc-KCTD17), myc-KCTD17 and GAPDH are shown by immunoblotting analysis.

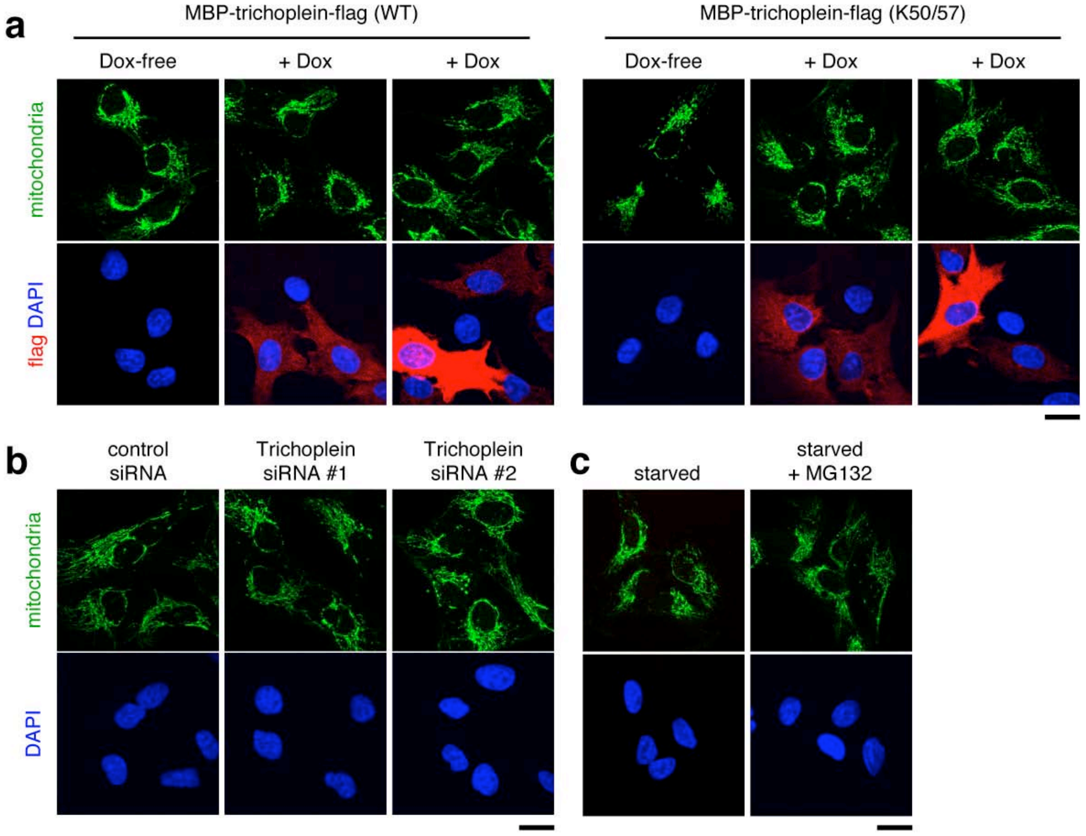

Supplementary Figure 10 | Effects of trichoplein overexpression, depletion, or stabilization on mitochondria morphology. (**a**) MBP-trichoplein-flag WT or K50/57R were overexpressed by Dox treatment in Tet-On RPE1 cells. (**b**) RPE1 cells were transfected with control or trichoplein (#1 and #2) siRNAs and cultured for 24 h. (**c**). RPE1 cells were subjected to 24 h serum starvation in the presence of DMSO (starved) or MG132 (starved + MG132). COX IV (a mitochondria maker, green), MBP-trichoplein-flag (red) and DNA (blue) are shown by indirect immunofluorescence. Bars indicate 10 μm.

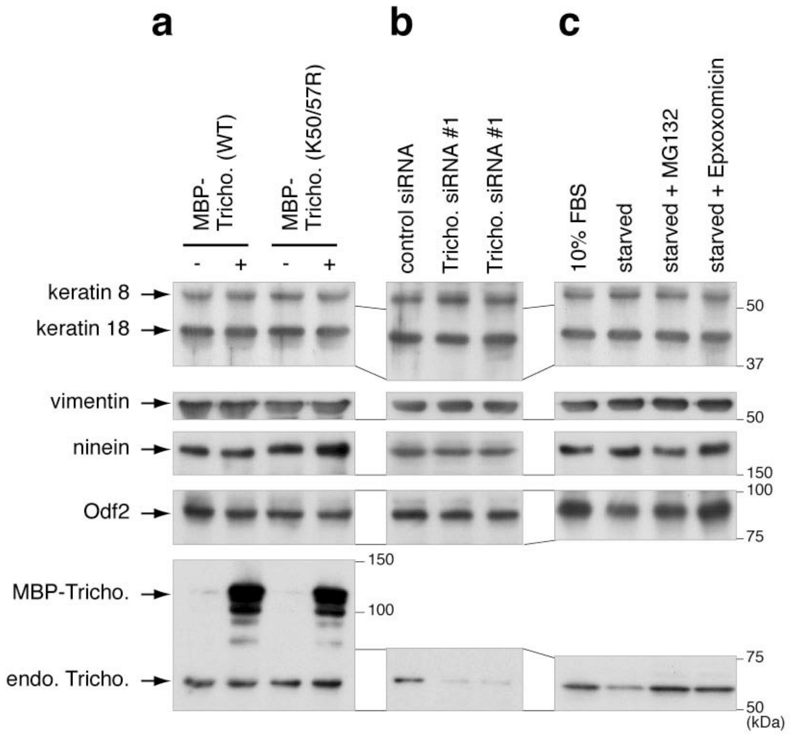

Supplementary Figure 11 | Effects of trichoplein overexpression, depletion, or stabilization on levels of its binding proteins. (**a**) MBP-trichoplein-flag WT or K50/57R were overexpressed by Dox treatment in Tet-On RPE1 cells. (**b**) RPE1 cells were transfected with control or trichoplein (#1 and #2) siRNAs and cultured for 24 h. (**c**). RPE1 cells were cultured in normal medium (10% FBS) or subjected to 24 h serum starvation in the presence of DMSO (starved), MG132 or Epoxomicin. Protein levels of keratin-8/18, vimentin, ninein, Odf2 and trichoplein are shown by immunoblotting analysis.

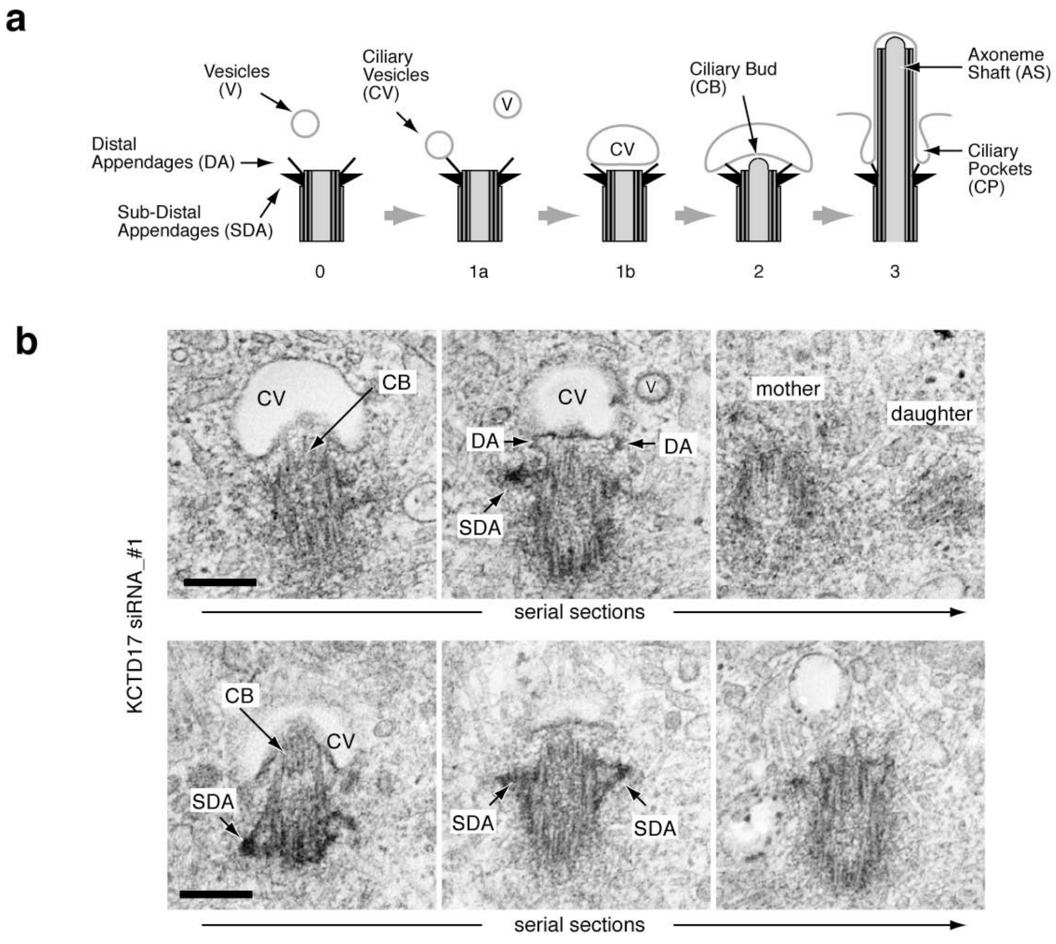

Supplementary Figure 12 | Ultrastructural analysis of KCTD17-depleted cells. (**a**) A schematic model illustrating the different stages of ciliogenesis, according to Sorokin[4,5]. The basal body is characterized by the presence of distal (DA) and subdistal appendages (SDA) (step 0). During early stages, ciliary vesicles (CV) dock at the basal body (step 1a). The CV increases in size by fusion with nearby secondary vesicles (V; step 1b), and become invaginated by the ciliary bud (CB) (step 2). Microtubule doublets start to elongate from the basal body giving rise to the axonemal shaft (AS) and the ciliary pocket (CP; step 3). Last, the elongated CV fuses with plasma membrane, and the cilium emerges in the extracellular environment. (**b**) TEM analysis of mother centrioles (serial sections) in RPE1 lacking KCTD17 (KCTD17 siRNA #1) subjected to 24 h serum starvation. Bars indicate 500 nm.

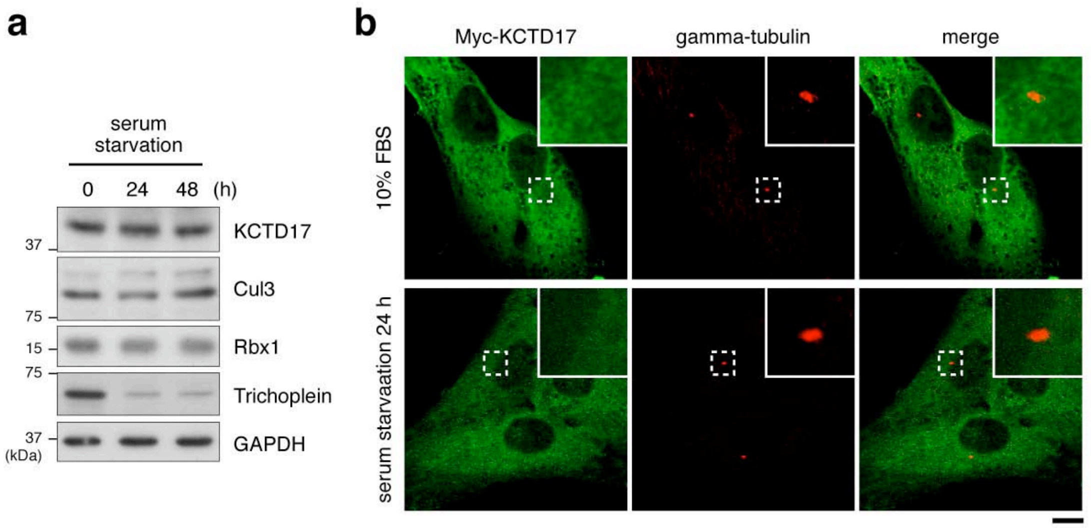

Supplementary Figure 13 | Protein level and localization of KCTD17 during ciliogenesis. (**a**) Immunoblotting analysis shows protein levels of KCTD17, Cul3, Rbx1, trichoplein and GAPDH in RPE1 cells cultured in normal medium (0 h) or subjected to serum starvation (24 or 48 h). (**b**) Tet-On RPE1 cells expressing myc-KCTD17 were cultured in normal medium (10% FBS) or subjected to serum starvation (24 h). Myc-KCTD17 (green), gamma-tubulin (red) and DNA (blue) are shown by indirect immunofluorescence. Bars indicate 10 μm.

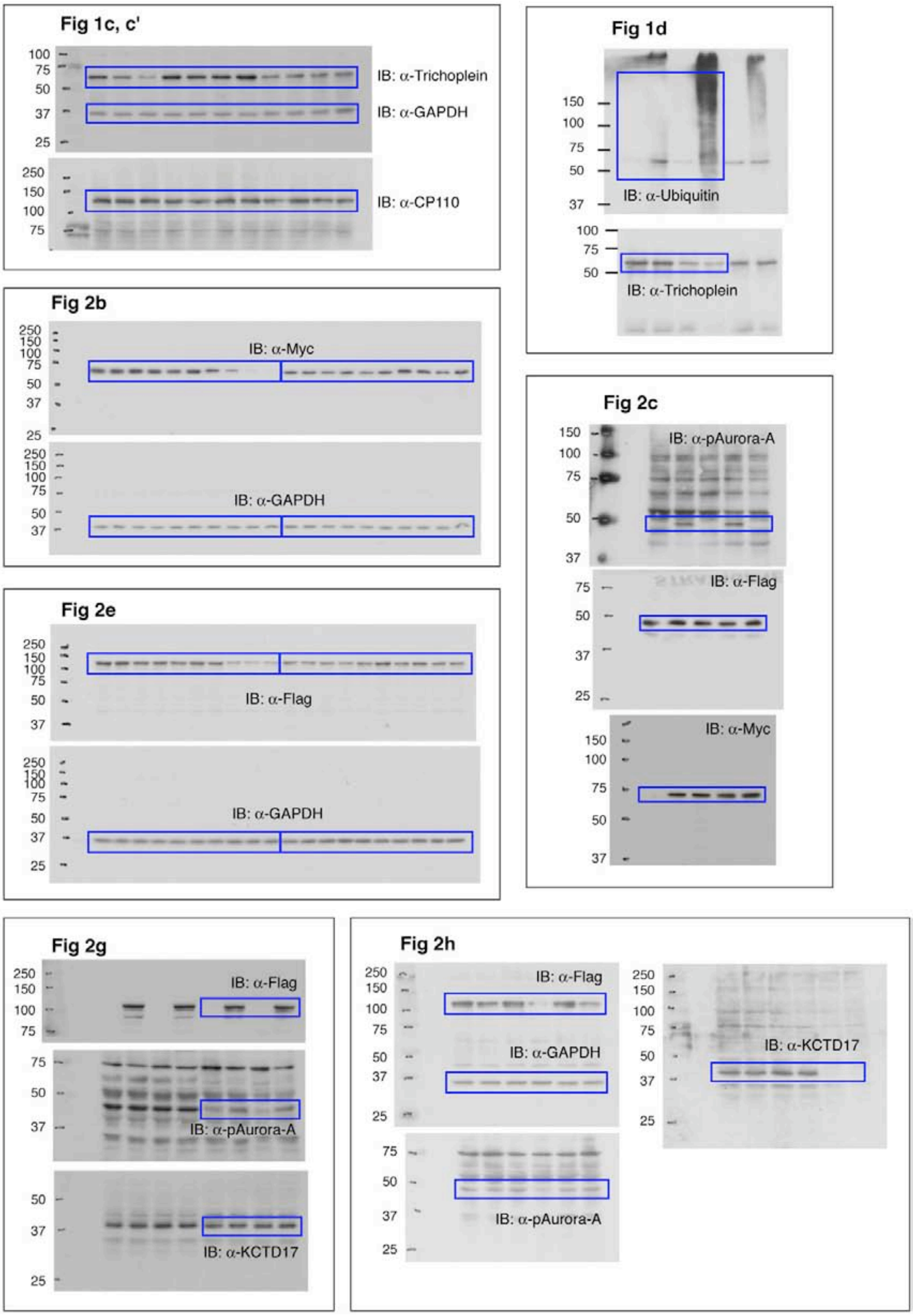

Supplementary Figure 14 | Uncropped versions of immunoblots referring to Fig. 1c, c' and d; and Fig. 2b, c, e, g and h.

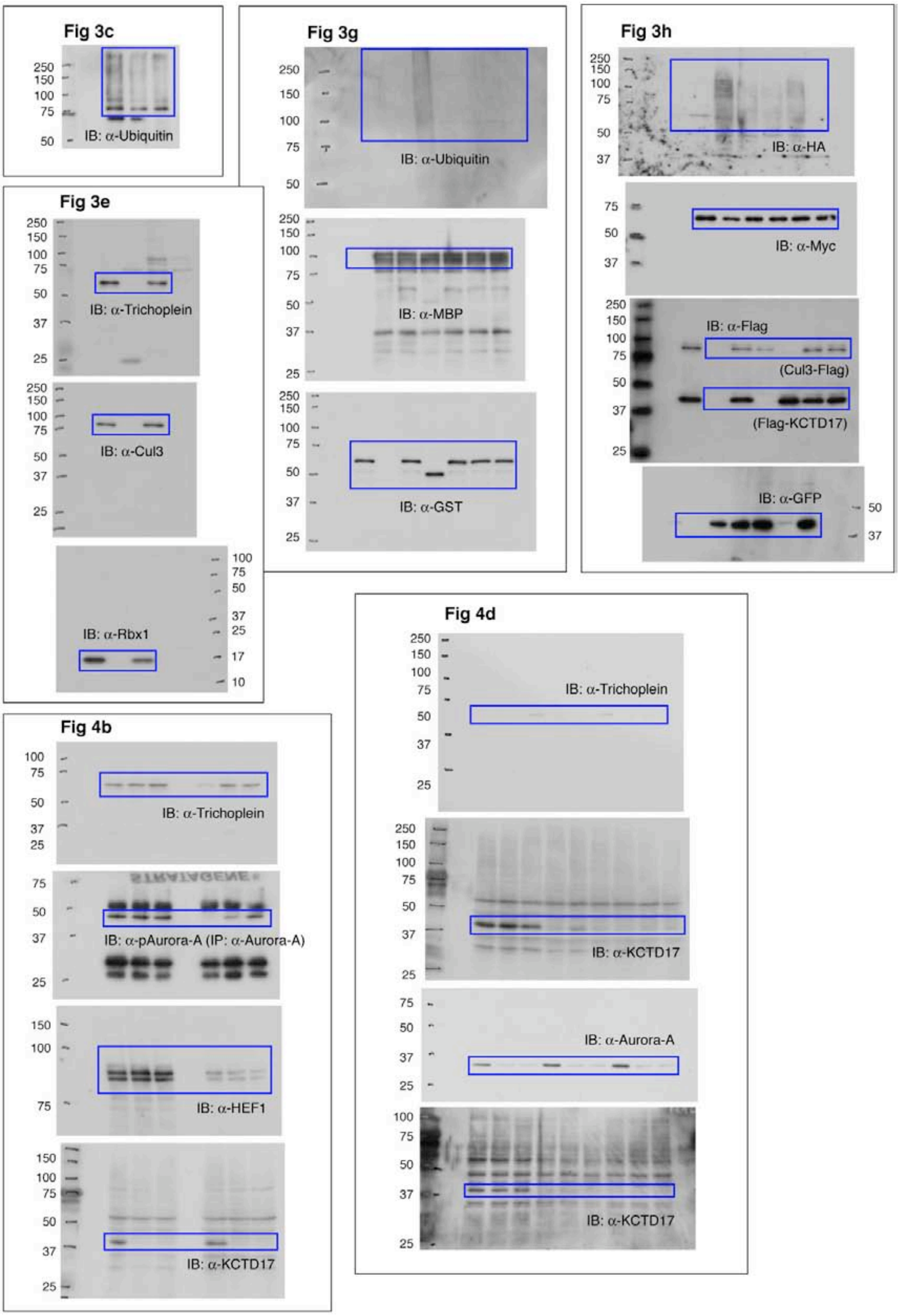

Supplementary Figure 15 | Uncropped versions of immunoblots referring to Fig. 3c, e, g and h; and Fig. 4b and d.

Supplementary Table 1. Human E3 ubiquitin ligase gene clones which were selected from HuPEX.

Gene symbol	HuPEX clones
ABTB1	FLJ25872AAAN FLJ27084AAAF FLJ33190AAAN FLJ96119AAAF
ABTB2	FLJ32301AAAN FLJ34241AAAN FLJ38313AAAN
AMBRA1	FLJ13135AAAN
AMFR	FLJ13812AAAN
ANAPC1	FLJ13745AAAF FLJ33101AAAN FLJ37208AAAN
ANAPC10	FLJ80998AAAF
ANAPC11	FLJ34041AAAN FLJ84203AAAF
ANAPC13	FLJ82510AAAF
ANAPC16	FLJ33728AAAN
ANAPC2	FLJ58169AAAN
ANAPC4	FLJ77597AAAN
ANAPC5	FLJ21961AAAN FLJ30217AAAN
ANAPC7	FLJ11747AAAN
ANKFY1	FLJ23744AAAN
ANKIB1	FLJ10317AAAN FLJ21822AAAN
ARIH1	FLJ93118AAAF
ARIH2	FLJ10938AAAN FLJ33921AAAN FLJ93973AAAF
ARMC5	FLJ13063AAAN FLJ55719AAAF
ASB10	FLJ30974AAAN FLJ43577AAAN
ASB12	FLJ39577AAAN
ASB13	FLJ11502AAAN FLJ13134AAAN
ASB15	FLJ43370AAAN
ASB16	FLJ30165AAAN
ASB17	FLJ25740AAAN
ASB18	FLJ41860AAAN
ASB2	FLJ11187AAAN FLJ32516AAAN FLJ35952AAAN FLJ96713AAAF
ASB3	FLJ10123AAAN FLJ10421AAAN FLJ31507AAAN FLJ54769AAAF
ASB4	FLJ82942AAAF
ASB5	FLJ25827AAAF FLJ39122AAAN
ASB6	FLJ20548AAAN FLJ54137AAAF
ASB7	FLJ22551AAAN
ASB8	FLJ21255AAAN FLJ43608AAAN FLJ55001AAAF
ASB9	FLJ20636AAAN
ATG16L1	FLJ10035AAAN FLJ14948AAAN FLJ22677AAAN FLJ23854AAAN FLJ41882AAAN
BACH1	FLJ94404AAAF
BARD1	FLJ51596AAAF FLJ95007AAAF
BCL6	FLJ98110SAAN
BCL6B	FLJ16548AAAN
BFAR	FLJ94525AAAF
BIRC2	FLJ93988AAAF
BIRC7	FLJ81925AAAF
BIRC8	FLJ82415WAAF
BRAP	FLJ50182AAAN
BRCA1	FLJ04047AAAN
BRWD1	FLJ11315AAAF
BTBD1	FLJ20724AAAN
BTBD10	FLJ38627AAAF FLJ76439AAAN
BTBD11	FLJ16416AAAN FLJ33957AAAN FLJ42845AAAN FLJ45068AAAN FLJ45362AAAF FLJ46588AAAF
BTBD16	FLJ25359AAAN
BTBD2	FLJ14575AAAN FLJ32256AAAF
BTBD3	FLJ50555AAAF
BTBD7	FLJ10648AAAN FLJ11891AAAN FLJ23774AAAF
BTBD8	FLJ81916AAAF
BTBD9	FLJ32945AAAN FLJ33611AAAF
BTRC	FLJ54002AAAF FLJ93878AAAF FLJ93955AAAF
CBL	FLJ09103AAAN
CBLB	FLJ36865AAAN FLJ41152AAAN
CBLC	FLJ81225AAAF
CBLL1	FLJ23109AAAN
CCIN	FLJ94364AAAF
CCNB1IP1	FLJ22580AAAF
CCNF	FLJ93902AAAF
CDC16	FLJ96326AAAF
CDC23	FLJ53431AAAF
CDC26	FLJ85201AAAF
CDC27	FLJ57525AAAN
CDCA3	FLJ34927AAAN FLJ93285AAAF
CGRRF1	FLJ96350AAAF
CHFR	FLJ10796AAAN FLJ14781AAAF FLJ30355AAAN FLJ40352AAAN
CIAO1	FLJ51287AAAN FLJ96640AAAF
CISH	FLJ94478AAAF
CNOT4	FLJ90012AAAN FLJ90190AAAN FLJ95362AAAN
CRBN	FLJ25462AAAN FLJ52785AAAF FLJ92952AAAF
CUL1	FLJ38844AAAN FLJ39186AAAN
CUL2	FLJ37898AAAN
CUL3	FLJ76286AAAN
CUL4A	FLJ95721AAAF
CUL4B	FLJ41694AAAN FLJ95982AAAF
CUL5	FLJ75330AAAN
CUL7	FLJ76468AAAN
CUL9	FLJ26138AAAN FLJ43238AAAN
DCAF10	FLJ90051AAAN
DCAF11	FLJ23716AAAN FLJ33074AAAN FLJ34538AAAN FLJ37159AAAN
DCAF12	FLJ20347AAAN
DCAF13	FLJ10831AAAN FLJ11012AAAF
DCAF15	FLJ35753AAAN
DCAF16	FLJ20280AAAN FLJ31554AAAN
DCAF17	FLJ13096AAAN
DCAF4	FLJ14839AAAN FLJ52764AAAF FLJ53137AAAF FLJ96563AAAF
DCAF5	FLJ36964AAAF FLJ83531AAAF
DCAF6	FLJ10122AAAF FLJ36651AAAN
DCAF7	FLJ54214AAAF
DCAF8	FLJ22828AAAN FLJ55296AAAF FLJ55437AAAF
DCST1	FLJ32785AAAN FLJ53894AAAF FLJ53895AAAF FLJ53937AAAF
DDA1	FLJ82470AAAF
DDB2	FLJ34321AAAN FLJ93771AAAF
DET1	FLJ10103AAAN FLJ30041AAAN
DIABLO	FLJ10537AAAN FLJ21115AAAN FLJ25049AAAN FLJ96714AAAF
DPF1	FLJ37313AAAN FLJ45263AAAN FLJ55156AAAF
DTL	FLJ10344AAAN FLJ10399AAAN FLJ14745AAAN FLJ20735AAAN FLJ53398AAAF
DTX2	FLJ13862AAAN FLJ22599AAAF FLJ59034AAAF
DTX3	FLJ34766AAAN FLJ37066AAAF
DTX3L	FLJ43096AAAF
DZIP3	FLJ13076AAAF
ECT2L	FLJ50042AAAN
EED	FLJ56640AAAF
ENC1	FLJ39259AAAN
ERCC8	FLJ32369AAAN FLJ52883AAAF FLJ56290AAAF FLJ95329AAAF
FANCL	FLJ10335AAAN FLJ51649AAAF
FBXL12	FLJ20188AAAN FLJ36441AAAN
FBXL13	FLJ38068AAAF FLJ40218AAAN
FBXL14	FLJ81572AAAF
FBXL15	FLJ82500AAAF
FBXL16	FLJ33735AAAF
FBXL17	FLJ44768AAAN
FBXL18	FLJ10776AAAN FLJ11467AAAN FLJ32480AAAN FLJ38075AAAN
FBXL19	FLJ45801AAAN
FBXL2	FLJ10409AAAN FLJ10576AAAN
FBXL20	FLJ41053AAAN FLJ92993AAAF
FBXL21	FLJ82790AAAF
FBXL22	FLJ82386AAAF
FBXL3	FLJ21831AAAN FLJ95240AAAF
FBXL4	FLJ93557AAAF
FBXL5	FLJ20146AAAN FLJ55999AAAF
FBXL8	FLJ11278AAAN
FBXO11	FLJ12673AAAN FLJ21824AAAN FLJ40415AAAN
FBXO15	FLJ36896AAAN
FBXO17	FLJ25205AAAN
FBXO18	FLJ14475AAAN FLJ14590AAAN FLJ16282AAAF FLJ38024AAAN FLJ96150AAAF
FBXO2	FLJ93487AAAF
FBXO21	FLJ10837AAAN FLJ90233AAAN
FBXO22	FLJ13986AAAN FLJ50132AAAN
FBXO25	FLJ52290AAAF FLJ60027AAAF FLJ84754AAAF
FBXO27	FLJ81115AAAF
FBXO28	FLJ10766AAAN FLJ50331AAAF
FBXO3	FLJ11081AAAN FLJ46597AAAN
FBXO30	FLJ41030AAAF FLJ42144AAAN FLJ94182AAAF
FBXO31	FLJ81555AAAF
FBXO32	FLJ32424AAAN
FBXO33	FLJ50090AAAF

Supplementary Table 1. (continued)

Gene symbol	HuPEX clones
FBXO34	FLJ20725AAAN FLJ35554AAAN FLJ35560AAAN
FBXO36	FLJ41090AAAN
FBXO38	FLJ13962AAAN FLJ83590AAAN
FBXO39	FLJ82180AAAF
FBXO4	FLJ10141AAAF FLJ14013AAAN
FBXO40	FLJ95177AAAF
FBXO41	FLJ45596AAAN
FBXO42	FLJ31036AAAF
FBXO44	FLJ30782AAAN FLJ50458AAAN FLJ50713AAAN
FBXO45	FLJ22044AAAN
FBXO5	FLJ30659AAAN FLJ95258AAAF
FBXO6	FLJ95911AAAF
FBXO7	FLJ30177AAAN FLJ55022AAAF FLJ76913AAAF
FBXO8	FLJ22666AAAF FLJ41746AAAF
FBXO9	FLJ11497AAAN FLJ37988AAAN
FBXW11	FLJ54601AAAF FLJ95926AAAF
FBXW12	FLJ40275AAAF
FBXW2	FLJ12422AAAN FLJ34541AAAN FLJ53609AAAF
	FLJ95158AAAF
FBXW4	FLJ95597AAAF
FBXW5	FLJ22428AAAN FLJ96847AAAF
FBXW7	FLJ11071AAAN FLJ55681AAAF
FBXW8	FLJ76330AAAN
FBXW9	FLJ41042AAAN FLJ52949AAAF
FEM1B	FLJ25754AAAN
G2E3	FLJ12805AAAN FLJ20333AAAN
GAN	FLJ38059AAAF
GMCL1	FLJ13057AAAN FLJ13199AAAN
GMCL1P1	FLJ96800AAAF
GNB2	FLJ32188AAAN FLJ84631AAAF
GRWD1	FLJ29021AAAN FLJ52246AAAF FLJ90195AAAN
	FLJ96709AAAF
GZF1	FLJ21794AAAN FLJ31597AAAN FLJ31915AAAF
	FLJ95435AAAF
HACE1	FLJ16076AAAN FLJ50472AAAN FLJ50673AAAN
HECTD1	FLJ21194AAAF FLJ43800AAAF
HECTD2	FLJ16050AAAF FLJ37306AAAN
HECTD3	FLJ31983AAAN FLJ32311AAAN FLJ34264AAAN
	FLJ39143AAAN
HECTD4	FLJ10510AAAN FLJ30092AAAF FLJ34154AAAN
HECW1	FLJ12617AAAN FLJ33480AAAN
HERC1	FLJ34305AAAF
HERC2	FLJ16724AAAN FLJ43239AAAN FLJ59185AAAF
HERC3	FLJ50787AAAN
HERC4	FLJ23155AAAN FLJ83643AAAN
HERC5	FLJ35836AAAN
HERC6	FLJ20637AAAN FLJ35084AAAN FLJ39849AAAN
HIC1	FLJ09113AAAN
HIC2	FLJ85428AAAN
HLTF	FLJ82789AAAF
HUWE1	FLJ20030AAAN FLJ32109AAAN
IBTK	FLJ44825AAAN
IPP	FLJ13818AAAN
ITCH	FLJ54369AAAF FLJ96205AAAF
IVNS1ABP	FLJ10411AAAN FLJ10763AAAN FLJ10962AAAN
	FLJ12958AAAN FLJ13061AAAN
KBTBD10	FLJ93742SAAN
KBTBD12	FLJ46299AAAN
KBTBD2	FLJ20904AAAN
KBTBD3	FLJ30685AAAF FLJ35674AAAF
KBTBD4	FLJ10450AAAF FLJ10887AAAN FLJ34288AAAN
KBTBD5	FLJ32015AAAN FLJ51312AAAN
KBTBD6	FLJ32071AAAN FLJ37876AAAN FLJ39289AAAN
KBTBD7	FLJ34025AAAN FLJ51290AAAN FLJ96667AAAF
KBTBD8	FLJ39321WAAN
KCNA2	FLJ82784AAAF
KCNA3	FLJ94663AAAF
KCNC2	FLJ37401AAAN
KCNC3	FLJ45584AAAN
KCND1	FLJ37112AAAF FLJ96049AAAF
KCNG1	FLJ46888AAAF
KCNG3	FLJ82413AAAF
KCNS2	FLJ83044AAAF
KCNS3	FLJ90607AAAN FLJ95252AAAF
KCNV1	FLJ53836AAAF FLJ94016AAAF
KCNV2	FLJ59225AAAF
KCTD1	FLJ75998AAAF FLJ82370AAAF

Gene symbol	HuPEX clones
KCTD10	FLJ32350AAAF
KCTD11	FLJ31665AAAN
KCTD12	FLJ46506AAAN
KCTD13	FLJ40088AAAN FLJ75386AAAF
KCTD14	FLJ94527AAAF
KCTD15	FLJ14995AAAN FLJ39524AAAN
KCTD17	FLJ12242AAAN
KCTD18	FLJ31322AAAN FLJ37818AAAN
KCTD19	FLJ52078AAAF FLJ52079AAAF
KCTD20	FLJ53295AAAF FLJ53683AAAF
KCTD21	FLJ54885AAAF
KCTD3	FLJ52085AAAF
KCTD4	FLJ76027AAAF FLJ81997AAAF
KCTD5	FLJ20040AAAN
KCTD6	FLJ14666AAAN FLJ90453AAAN
KCTD7	FLJ32069AAAN
KCTD8	FLJ41353AAAN
KCTD9	FLJ20038AAAN
KDM2A	FLJ46431AAAN FLJ51294AAAN
KDM2B	FLJ14534AAAN FLJ14786AAAN FLJ40614AAAN
	FLJ45399AAAF FLJ55590AAAF FLJ90237AAAN
KEAP1	FLJ31642AAAN
KIAA0317	FLJ55279AAAF
KLHDC5	FLJ38086AAAN
KLHL1	FLJ30047AAAN FLJ52306AAAF
KLHL10	FLJ32662AAAN FLJ51379AAAN
KLHL11	FLJ10572AAAN FLJ51370AAAN
KLHL12	FLJ14350AAAN FLJ14750AAAF
KLHL13	FLJ10262AAAF FLJ16227AAAF FLJ43366AAAN
	FLJ55342AAAF FLJ55461AAAF FLJ55504AAAF
KLHL15	FLJ32736AAAN
KLHL17	FLJ45680AAAF FLJ50001AAAF
KLHL18	FLJ13703AAAN
KLHL2	FLJ21636AAAN FLJ96418AAAF
KLHL20	FLJ10568AAAN
KLHL21	FLJ90824AAAN
KLHL22	FLJ14360AAAN
KLHL24	FLJ20059AAAF FLJ22673AAAN FLJ25796AAAN
KLHL25	FLJ12587AAAN FLJ32101AAAN FLJ34912AAAF
	FLJ96608AAAF
KLHL26	FLJ11078AAAN
KLHL28	FLJ20081AAAF
KLHL29	FLJ14106AAAN
KLHL3	FLJ40871AAAN FLJ50667AAAN FLJ52387AAAF
	FLJ95562AAAF
KLHL30	FLJ43374AAAN
KLHL32	FLJ30730AAAN FLJ50156AAAF FLJ52328AAAF
KLHL34	FLJ34960AAAN
KLHL36	FLJ12543AAAN FLJ16270AAAN
KLHL4	FLJ12653AAAN FLJ12995AAAN FLJ31895AAAF
KLHL5	FLJ10836AAAN FLJ10974AAAN FLJ11312AAAN
KLHL6	FLJ39806AAAF FLJ40657AAAN FLJ95278AAAF
KLHL7	FLJ31828AAAF FLJ52310AAAF FLJ52332AAAF
	FLJ53164AAAF FLJ54026AAAF FLJ54973AAAF
	FLJ55340AAAF
KLHL8	FLJ46304AAAN
KLHL9	FLJ13568AAAN FLJ34396AAAN
LGALS3BP	FLJ25047AAAN FLJ31415AAAF FLJ53427AAAF
	FLJ53478AAAF FLJ53509AAAF FLJ54583AAAF
LMO7	FLJ53272AAAF
LNX1	FLJ32261AAAN
LNX2	FLJ23932AAAN FLJ38000AAAF FLJ93928AAAF
LONRF1	FLJ23749AAAN FLJ54859AAAF
LONRF2	FLJ45273AAAN
LONRF3	FLJ22612AAAN FLJ34458AAAN FLJ40322AAAN
LRR1	FLJ54648AAAF
LRRC29	FLJ38782AAAN FLJ96712AAAF
LRSAM1	FLJ31641AAAN FLJ31743AAAN FLJ34270AAAN
	FLJ39775AAAN
LTN1	FLJ13437AAAN
LZTR1	FLJ45137AAAN FLJ53345AAAF FLJ94057AAAF
MARCH1	FLJ20668AAAN FLJ21352AAAN FLJ76212AAAF
MARCH10	FLJ16660AAAF FLJ35757AAAN
MARCH2	FLJ26653AAAN
MARCH3	FLJ76009AAAF FLJ82282AAAF
MARCH5	FLJ20445AAAF

Supplementary Table 1. (continued)

Gene symbol	HuPEX clones
MARCH6	FLJ10108AAAN FLJ54336AAAF FLJ55749AAAF FLJ95538AAAF
MARCH8	FLJ83990AAAF FLJ93862AAAF
MARCH9	FLJ36578AAAF FLJ94564AAAF
MDM2	FLJ75260WAAN FLJ81856WAAF
MDM4	FLJ51436AAAF FLJ85796AAAN
MEX3A	FLJ43493AAAN
MEX3B	FLJ16544AAAN FLJ36826AAAN
MEX3C	FLJ38871AAAN FLJ92328AAAF
MGRN1	FLJ82812AAAF
MIB1	FLJ33947AAAF FLJ90676AAAF
MIB2	FLJ16279AAAN FLJ16491AAAN FLJ25919AAAN FLJ34291AAAN FLJ38595AAAN FLJ38976AAAF FLJ39787AAAN FLJ46290AAAN FLJ46712AAAN
MID1	FLJ21534AAAN FLJ96053AAAF
MID2	FLJ81560AAAF
MKRN1	FLJ21334AAAN FLJ23484AAAF FLJ45086AAAF FLJ96622AAAF
MKRN2	FLJ33999AAAN FLJ52346AAAF
MKRN3	FLJ82266AAAF
MLL3	FLJ12625AAAN
MSL2	FLJ10546AAAN FLJ54913AAAF
MUL1	FLJ12875AAAN FLJ52155AAAF FLJ55316AAAF
MYCBP2	FLJ21597AAAF FLJ21646AAAN
MYLIP	FLJ23811AAAN FLJ95492AAAF
MYNN	FLJ11584AAAN FLJ52196AAAF
NEDD4	FLJ54469AAAF
NEDD4L	FLJ04160AAAN
NEURL	FLJ81570AAAF
NEURL2	FLJ30259AAAN
NEURL3	FLJ54814AAAF
NFXL1	FLJ16294AAAF
NLE1	FLJ10458AAAN
NOSIP	FLJ35125AAAN FLJ54756AAAF FLJ75703AAAF FLJ84625AAAF
NSMCE1	FLJ32233AAAN FLJ96768AAAF
NUP43	FLJ23731AAAF FLJ38675AAAN FLJ53749AAAF FLJ54281AAAF
OSTM1	FLJ26344AAAN FLJ90531AAAN
PAFAH1B1	FLJ51164AAAF FLJ52123AAAF FLJ93559AAAN
PARK2	FLJ82639AAAF
PATZ1	FLJ83525AAAN
PCGF1	FLJ43754AAAN
PCGF2	FLJ81738AAAN
PCGF3	FLJ32562AAAN FLJ43813AAAN FLJ54810AAAF FLJ83855WAAF
PCGF5	FLJ51751AAAF
PCGF6	FLJ14979AAAN FLJ84231AAAF
PDZRN3	FLJ54738AAAF FLJ54746AAAF FLJ90092AAAN
PDZRN4	FLJ16796AAAN FLJ37371AAAN FLJ52105AAAF
PEX10	FLJ42826AAAN FLJ84836AAAF
PEX12	FLJ93019AAAF
PEX2	FLJ93460AAAN
PHF7	FLJ82033AAAF
PHIP	FLJ20705AAAF FLJ90643AAAN
PJA1	FLJ11830AAAF FLJ39292AAAN
PJA2	FLJ80392AAAN
PML	FLJ04040AAAN FLJ04040SAAN FLJ23218AAAN FLJ40881AAAF
POC1B	FLJ14923AAAN FLJ41111AAAN FLJ58572AAAF FLJ90291AAAN
PPIL2	FLJ45326AAAN FLJ84832AAAN
PRPF19	FLJ81036AAAF
PWP1	FLJ51512AAAF FLJ51513AAAF
RAB40A	FLJ32297AAAN
RAB40B	FLJ81994AAAF
RABGEF1	FLJ10840AAAN FLJ32302AAAN FLJ34077AAAF FLJ45891AAAN FLJ55772AAAF
RAD18	FLJ13013AAAN
RAG1	FLJ82750AAAF
RBBP4	FLJ50184AAAF FLJ92946AAAF
RBBP5	FLJ83102AAAF
RBBP6	FLJ04037AAAN FLJ23301AAAN
RBBP7	FLJ34592AAAN
RBCK1	FLJ81936AAAF
RBX1	FLJ96824AAAF
RC3H1	FLJ04046AAAN FLJ16661AAAF FLJ36182AAAN

Gene symbol	HuPEX clones
RC3H2	FLJ04013AAAN FLJ20301AAAF FLJ20713AAAN FLJ23389AAAN
RCBTB1	FLJ10716AAAN FLJ33488AAAN FLJ39335AAAN
RCBTB2	FLJ12948AAAF FLJ16324AAAN FLJ54251AAAF FLJ96802AAAF
RCHY1	FLJ34182AAAN
RFFL	FLJ35793AAAN
RFPL2	FLJ31437AAAN
RFPL3	FLJ40419AAAF
RFPL4B	FLJ16581AAAN
RFWD2	FLJ10416AAAN FLJ95614AAAF
RFWD3	FLJ10520AAAN FLJ12611AAAN FLJ96902AAAF
RHOBTB1	FLJ90648AAAN
RHOBTB2	FLJ76193AAAN
RHOBTB3	FLJ13559AAAN
RING1	FLJ51343AAAN FLJ76639AAAN
RLIM	FLJ10472AAAN FLJ95628AAAF
RNF10	FLJ36692AAAN FLJ40488AAAN FLJ44288AAAF FLJ96629AAAF
RNF103	FLJ16725AAAN FLJ94902AAAF
RNF11	FLJ93630AAAF
RNF111	FLJ16278AAAF FLJ16671AAAN FLJ38008AAAN
RNF112	FLJ52256AAAF
RNF113A	FLJ95648AAAF
RNF113B	FLJ81147AAAF
RNF114	FLJ34657AAAN FLJ54192AAAF FLJ96725AAAF
RNF115	FLJ75672AAAF FLJ82335AAAF
RNF121	FLJ11099AAAN FLJ13077AAAN FLJ37189AAAN
RNF122	FLJ12526AAAN
RNF123	FLJ12565AAAN FLJ23315AAAF FLJ33313AAAN FLJ39034AAAN FLJ44935AAAN
RNF126	FLJ20552AAAN
RNF128	FLJ23684AAAN FLJ44589AAAF
RNF13	FLJ33319AAAN FLJ33452AAAF
RNF130	FLJ84413AAAF FLJ93572AAAF
RNF133	FLJ25658AAAF
RNF135	FLJ16075AAAN FLJ16196AAAN
RNF138	FLJ13517AAAN FLJ96873AAAF
RNF139	FLJ10740AAAN FLJ21390AAAN FLJ25653AAAN
RNF14	FLJ13636AAAF FLJ13822AAAN FLJ25139AAAN
RNF141	FLJ75906AAAF
RNF144A	FLJ80777AAAF
RNF144B	FLJ39513AAAN
RNF145	FLJ25936AAAN FLJ44310AAAN FLJ55465AAAF FLJ55623AAAF FLJ60012AAAF FLJ90620AAAN
RNF146	FLJ14530AAAN FLJ14652AAAN FLJ14870AAAF
RNF148	FLJ25387AAAN FLJ25788AAAN
RNF149	FLJ26288AAAN FLJ90504AAAN FLJ90660AAAN
RNF150	FLJ27010AAAN
RNF151	FLJ82945AAAF
RNF152	FLJ16292AAAN FLJ39176AAAN
RNF157	FLJ31387AAAN FLJ34148AAAN
RNF165	FLJ16418AAAN FLJ45559AAAN
RNF166	FLJ32544AAAN FLJ32639AAAN FLJ84091AAAF
RNF167	FLJ21676AAAN
RNF168	FLJ35794AAAN
RNF17	FLJ11045AAAN
RNF170	FLJ14842AAAN FLJ33545AAAN
RNF175	FLJ34190AAAN FLJ84045AAAF
RNF180	FLJ33437AAAF
RNF181	FLJ80975AAAF
RNF182	FLJ33257AAAN FLJ96594AAAF
RNF183	FLJ34621AAAN
RNF185	FLJ53598AAAF FLJ84293AAAF
RNF186	FLJ20225AAAN
RNF19A	FLJ10912AAAN FLJ13393AAAN FLJ23417AAAN FLJ33137AAAF
RNF19B	FLJ16569AAAN FLJ90005AAAF
RNF2	FLJ34255AAAN FLJ95665AAAN
RNF20	FLJ11189AAAN FLJ12238AAAN FLJ12470AAAN FLJ95178AAAF
RNF207	FLJ32096AAAN FLJ45800AAAN FLJ46380AAAN FLJ54599AAAF
RNF208	FLJ81973AAAF
RNF212	FLJ38841AAAN FLJ38841SAAN

Supplementary Table 1. (continued)

Gene symbol	HuPEX clones
RNF213	FLJ13051AAAN FLJ13809AAAN FLJ22023AAAF FLJ22023WAAN FLJ22261AAAN FLJ22385AAAN FLJ45432AAAF FLJ46021AAAN
RNF214	FLJ54868AAAF
RNF215	FLJ59014AAAF
RNF216	FLJ10054AAAF FLJ21134AAAN FLJ41641AAAN FLJ95092AAAF
RNF217	FLJ16403AAAN
RNF219	FLJ13449AAAN FLJ25774AAAN
RNF220	FLJ10597AAAN FLJ31862AAAN FLJ42800AAAF FLJ52472AAAF FLJ54701AAAF FLJ54815AAAF
RNF24	FLJ81616AAAF
RNF25	FLJ13906AAAN FLJ75387AAAF
RNF26	FLJ16070AAAN FLJ91063AAAN
RNF31	FLJ10111AAAN FLJ16199AAAF
RNF34	FLJ21786AAAF FLJ39675AAAN FLJ52837AAAF
RNF38	FLJ21343AAAN FLJ36161AAAN FLJ40334AAAN
RNF4	FLJ46157AAAF
RNF40	FLJ14500AAAN FLJ16291AAAN FLJ91219AAAN
RNF41	FLJ50872AAAF FLJ51733AAAF FLJ95687AAAF
RNF43	FLJ20315AAAN FLJ50102AAAN FLJ51697AAAF
RNF44	FLJ45771AAAN
RNF5	FLJ51221AAAF FLJ92125AAAF
RNF6	FLJ53858AAAF FLJ92781AAAN
RNF7	FLJ92255AAAF
RNF8	FLJ12013AAAN FLJ50824AAAN
RNFT1	FLJ25783AAAN
RNFT2	FLJ14627AAAN FLJ35845AAAN
RSPRY1	FLJ14643AAAN FLJ24006AAAF FLJ42610AAAN
SCAF11	FLJ94773AAAF
SF3B3	FLJ10435AAAN FLJ90452AAAF
SH3RF1	FLJ21602AAAN
SH3RF2	FLJ23654AAAF FLJ25317AAAN
SHKBP1	FLJ41701AAAN FLJ53408AAAF FLJ55912AAAF FLJ90576AAAN FLJ93157AAAF
SHPRH	FLJ27258AAAN FLJ45012AAAN
SIAH1	FLJ08065AAAN FLJ37344AAAN FLJ94493AAAF
SIAH2	FLJ30728AAAN FLJ83477AAAF
SKP1	FLJ08087AAAN
SKP2	FLJ53740AAAF
SLX4	FLJ16318AAAN FLJ38092AAAN
SMU1	FLJ10805AAAN FLJ54259AAAF
SMURF2	FLJ82924AAAF FLJ85600SAAN
SNRNP40	FLJ41108AAAN FLJ56825AAAF FLJ90035AAAN FLJ94417AAAF
SOCS1	FLJ45719AAAN
SOCS2	FLJ08186AAAN FLJ52461AAAF FLJ93661AAAF
SOCS3	FLJ94944AAAF
SOCS4	FLJ42089AAAN FLJ45366AAAN
SOCS5	FLJ51885AAAF
SOCS7	FLJ07010AAAN
SPOP	FLJ51096AAAN FLJ51172AAAF FLJ53827AAAF FLJ93086AAAF
SPOPL	FLJ11857AAAF FLJ53775AAAF FLJ85173AAAF
SPSB1	FLJ22393AAAN FLJ26545AAAN
SPSB2	FLJ55414AAAF
SPSB3	FLJ25121AAAN FLJ51375AAAN
SPSB4	FLJ31805AAAF
STUB1	FLJ81025AAAN
SYVN1	FLJ44964AAAN
TBL1X	FLJ82316AAAF
TBL1XR1	FLJ22093AAAN FLJ55712AAAF
TCEB1	FLJ25160AAAN
TCEB2	FLJ83036AAAF
TCEB3	FLJ93561AAAF
TLE1	FLJ50374AAAF FLJ59241AAAF
TLE2	FLJ41188AAAN FLJ85685AAAN
TLE3	FLJ10396AAAF FLJ39460AAAN
TNFAIP1	FLJ50737AAAN FLJ92717AAAF
TNFAIP3	FLJ93305AAAF
TOR1AIP2	FLJ83828WAAF
TRAF2	FLJ30124AAAN FLJ53315AAAF FLJ54037AAAF FLJ75198AAAN
TRAF4	FLJ81684AAAN
TRAF5	FLJ44538AAAF FLJ55439AAAF
TRAF6	FLJ75865AAAN FLJ82127AAAF
TRAF7	FLJ33305AAAF
TRAIP	FLJ08192AAAN
TRIM11	FLJ40506AAAN FLJ56023AAAF FLJ90142AAAF FLJ90385AAAN FLJ95361AAAF
TRIM13	FLJ10944AAAN FLJ95311AAAF
TRIM15	FLJ82219AAAN
TRIM17	FLJ30864AAAN
TRIM2	FLJ82555AAAF
TRIM21	FLJ81065AAAF
TRIM22	FLJ51040AAAN FLJ51049AAAN FLJ51115AAAN FLJ76655AAAN
TRIM24	FLJ45687AAAF FLJ90825AAAF
TRIM26	FLJ16483AAAN
TRIM27	FLJ81103AAAF
TRIM28	FLJ29029AAAN FLJ80047AAAN FLJ94025AAAF
TRIM3	FLJ16135AAAN FLJ44731AAAF FLJ55875AAAF
TRIM31	FLJ81974AAAF
TRIM32	FLJ85452AAAF
TRIM33	FLJ13372AAAN FLJ32925AAAN FLJ55940AAAF
TRIM34	FLJ14970AAAN
TRIM36	FLJ25450AAAF
TRIM37	FLJ21995AAAF
TRIM38	FLJ93754AAAF
TRIM4	FLJ35573AAAN
TRIM40	FLJ82349AAAF
TRIM41	FLJ14695AAAN
TRIM42	FLJ40097AAAN
TRIM43	FLJ81122AAAF
TRIM45	FLJ13181AAAN
TRIM46	FLJ23229AAAN FLJ38839AAAF FLJ46260AAAN
TRIM47	FLJ82606AAAF
TRIM48	FLJ80963AAAF
TRIM5	FLJ14687AAAN FLJ23783AAAN
TRIM50	FLJ32804AAAN
TRIM51	FLJ80994AAAF
TRIM52	FLJ30240AAAN
TRIM55	FLJ33991AAAN FLJ34409AAAN
TRIM56	FLJ35608AAAF FLJ83690AAAN FLJ90774AAAF
TRIM58	FLJ38869AAAN
TRIM6	FLJ14758AAAN FLJ51024AAAN
TRIM60	FLJ35882AAAN
TRIM62	FLJ10759AAAN FLJ16558AAAN FLJ51302AAAN
TRIM63	FLJ32380AAAN FLJ50813AAAN FLJ83113WAAF
TRIM67	FLJ44831AAAN
TRIM68	FLJ10369AAAN FLJ12861AAAN FLJ31961AAAN FLJ54177AAAF
TRIM69	FLJ82288AAAF
TRIM7	FLJ95773AAAF
TRIM72	FLJ16664AAAN FLJ33376AAAN
TRIM73	FLJ84145AAAF
TRIM8	FLJ81566AAAF
TRIM9	FLJ30826AAAN
TRIML1	FLJ36180AAAN
TRIP12	FLJ52804AAAF
TRPC4AP	FLJ50256AAAN FLJ50335AAAN FLJ51744AAAF FLJ96658AAAF
TSPAN17	FLJ82872AAAF
TTC3	FLJ53531AAAF FLJ82969AAAF
UBE3A	FLJ26981AAAN FLJ77551AAAN FLJ77614AAAN FLJ80585SAAN
UBE3B	FLJ45294AAAN FLJ51547AAAF FLJ51548AAAF
UBE3C	FLJ45347AAAF
UBE4A	FLJ51394AAAN
UBE4B	FLJ78424AAAN
UBOX5	FLJ12382AAAN
UBR1	FLJ45570AAAN
UBR2	FLJ23295AAAN FLJ23345AAAN FLJ33555AAAN FLJ43807AAAN FLJ45378AAAN
UBR3	FLJ45053AAAN FLJ45646AAAN FLJ53501AAAF
UBR4	FLJ10371AAAF FLJ12260AAAN FLJ12511AAAN FLJ12862AAAN FLJ13764AAAF FLJ31656AAAN FLJ41863AAAN FLJ53474AAAF FLJ53484AAAF
UBR5	FLJ11310AAAN
UHRF1	FLJ21925AAAN FLJ95411AAAF
UHRF2	FLJ16243AAAN
UNKL	FLJ12623AAAN FLJ23360AAAN
VHL	FLJ51515AAAF FLJ96920AAAF
VPRBP	FLJ13177AAAN

Supplementary Table 1. (continued)

Gene symbol	HuPEX clones	Gene symbol	HuPEX clones
VPS11	FLJ13217AAAF FLJ22359AAAF FLJ32139AAAN	ZBTB33	FLJ92632AAAF
VPS18	FLJ82481AAAF	ZBTB37	FLJ32748AAAN FLJ84795AAAF
VPS41	FLJ23195AAAN FLJ23745AAAN FLJ95380AAAF	ZBTB38	FLJ22332AAAN FLJ36882AAAN
VPS8	FLJ12883AAAN	ZBTB39	FLJ76063AAAN
WDR12	FLJ10881AAAN FLJ12719AAAN FLJ51286AAAN	ZBTB4	FLJ16429AAAF FLJ16715AAAN FLJ23490AAAN
WDR26	FLJ04162AAAN FLJ21016AAAF	ZBTB40	FLJ37954AAAN FLJ95610AAAF
WDR5	FLJ20545AAAN	ZBTB41	FLJ36199AAAN FLJ46708AAAN
WDR53	FLJ81816WAAF FLJ94793AAAF	ZBTB43	FLJ22470AAAN
WDR59	FLJ12270AAAF FLJ33997AAAF FLJ54513AAAF	ZBTB44	FLJ21006AAAN FLJ83953AAAN FLJ95555AAAF
WDR5B	FLJ11287AAAN FLJ96167AAAF	ZBTB45	FLJ14486AAAN FLJ26546AAAN
WDR61	FLJ21101AAAN	ZBTB46	FLJ16656AAAN
WDR76	FLJ12973AAAN	ZBTB47	FLJ45122AAAN
WDR82	FLJ23710AAAN FLJ25569AAAN FLJ41866AAAF	ZBTB48	FLJ93203AAAF
WDSUB1	FLJ26473AAAN FLJ36175AAAN	ZBTB49	FLJ38559AAAN FLJ45653AAAF
WDTC1	FLJ10872AAAN FLJ13039AAAN FLJ13716AAAN	ZBTB5	FLJ82547AAAN
WHSC1	FLJ23286AAAN FLJ77008AAAN FLJ84428SAAN	ZBTB6	FLJ77379AAAN
	FLJ85606SAAN	ZBTB7A	FLJ82903AAAN FLJ85799WAAN
WSB1	FLJ90436AAAN	ZBTB7B	FLJ55472AAAF
WSB2	FLJ59692AAAF FLJ83086AAAF	ZBTB8A	FLJ90065AAAN
WWP1	FLJ82196AAAN	ZBTB8B	FLJ35007AAAN
WWP2	FLJ44359AAAN FLJ55648AAAF FLJ93210AAAF	ZBTB9	FLJ16072AAAN FLJ94135AAAF
XIAP	FLJ94517AAAF	ZC3HC1	FLJ10455AAAN FLJ12311AAAN
ZBTB1	FLJ82307AAAF	ZFAND3	FLJ13222AAAN
ZBTB10	FLJ12752AAAN FLJ32351AAAN	ZFAND4	FLJ33385AAAN FLJ40185AAAF FLJ95171AAAF
ZBTB11	FLJ13426AAAN FLJ93302AAAF	ZFAND5	FLJ22129AAAN
ZBTB12	FLJ81360AAAF	ZFAND6	FLJ93032AAAF
ZBTB16	FLJ82076AAAF	ZFP161	FLJ93740AAAF
ZBTB17	FLJ50396AAAN FLJ50647AAAF FLJ50726AAAN	ZFPL1	FLJ52460AAAF FLJ76912AAAN
	FLJ54401AAAF FLJ96187AAAF	ZNF131	FLJ32781AAAF FLJ55375AAAF
ZBTB2	FLJ35567AAAN FLJ84026AAAN	ZNF238	FLJ84916AAAN
ZBTB20	FLJ26458AAAN FLJ96336AAAF	ZNF295	FLJ82852AAAF
ZBTB22	FLJ38659AAAN FLJ96856AAAF	ZNF645	FLJ25735AAAF
ZBTB25	FLJ40862AAAN	ZNRF1	FLJ58842AAAF
ZBTB26	FLJ25354AAAN FLJ32883AAAN	ZNRF3	FLJ22057AAAN FLJ39078AAAN
ZBTB3	FLJ23392AAAN	ZNRF4	FLJ25856AAAF
ZBTB32	FLJ82608AAAF	ZSWIM2	FLJ46125AAAN

Supplementary Table 1 | Human E3 ubiquitin ligase gene clones which were selected from HuPEX. We selected 744 genes to be human E3 ubiquitin ligase by a keyword search of the Human Gene and Protein Database (HGPD)[6], Swiss-Prot database and few papers[7-9]. We have 622 genes to these 744 genes, which were corresponding to 1,172 HuPEX clones containing variant clones.

Supplementary Table 2. List of antibodies.

Antibody	Clone/catalog #	Distribution source	IB	IF
acetylated tubulin	6-11B-1	Sigma-Aldrich	-	x100
Aurora-A	1G4	Cell Signaling Technology	x500*	-
Aurora-A-pT288	ab58494	Abcam	-	x100**
Aurora-A-pT288	C39D8	Cell Signaling Technology	x1,000*	-
BrdU	BU-1	Millipore	-	x5
COX IV	3E 11	Cell Signaling Technology	-	x250
CP110	12780-1-AP	ProteinTech	x2,500*	-
Cul3 (cullin 3)	Clone 3	Santa Cruz Biotechnlogy	x2,000	-
cyclin A	611268	BD Transduction	-	x200
DYKDDDDK (Flag)	1E 6	Wako Chemicals	x2,500	-
Flag	M2	Sigma-Aldrich	x10,000	-
gamma tubulin	GTU-88	Sigma-Aldrich	-	x1,000
gamma tubulin	T3359	Sigma-Aldrich	-	x500
GAPDH	14C10	Cell Signaling Technology	x2,000	-
GFP	Clones 7.1 & 13.1	Roche	x2,000	-
GST	B-14	Santa Cruz Technology	x2,000	-
HA	12CA5	Roche	x2,000	-
HA	561-7	MBL	x2,000	-
HEF1 (NEDD9)	2G9	Cell Signaling Technology	x1,000*	-
KCTD17	Rabbit polyAb	Home-made	x20,000*	-
cytokeratin	C2562	Sigma-Aldrich	x25,000	-
Ki-67	MIB-1	Dako	-	x20,000
MBP	1G12	MBL	x2,000	-
Myc	MC045	Nacalai tesque	x2,000	-
Myc	9B11	Millipore	x2,000	x2,000
Myc	4A6	Millipore	x2,000	-
ninein	A301-504A	Bethyl	x2,000*	-
Odf-2	HAP001874	Sigma-Aldrich	x1,000*	x100**
Rbx1 (Roc1)	EPR6850[B]	Abcam	x2,500	-
trichoplein	Rabbit polyAb	Home-made	x2,000*	x200
ubiquitin	P4D1	Cell Signaling Technology	x1,000*	-
vimentin	D21H3	Cell Signaling Technology	x1,000	-

* diluted in Can Get Signal® immunoreaction Enhanser Solution (TOYOBO, Osaka, Japan)

** diluted in Can Get Signal® immunostain Solution A (TOYOBO, Osaka, Japan)

Supplementary Table 2 | List of antibodies. The list shows antibodies with source and conditions of immunoblotting and indirect immunofluorescence.

Supplementary Table 3. List of siRNAs

**siRNA**	**Target sequence**
Negatvie control	AATTCTCCGAACGTGTCACGT
Tichoplein #1	AAGGCAGAATGGAGCTCTAAA
Tichoplein #2	TCCCAGCGCATTCCTTTGCAA
Aurora-A #1	TCCCAGCGCATTCCTTTGCAA
Aurora-A #2	CAGGGCTGCCATATAACCTGA
KCTD17 #1	CACGCAAATGGTCTCCACCAT
KCTD17 #2	CCGCATCATCAAAGACCGGAT
KCTD17 #3	CCCGGGCCTGAGAAGGAAGAA
KCTD17 #4	AACACTGTCTTTGCAAATGGA
CUL3 #1	AACAACTTTCTTCAAACGCTA
CUL3 #2	AACGTCTACAATTTGGGATTA
RBX1 #1	AAGAAGCGCTTTGAAGTGAAA
RBX1 #2	CTGCTGTTACCTAATTACAAA
RC3H2 #1	ACCCAAGACTCCTGTAAGTAA
RC3H2 #2	CCCGTACAGGTTACCATACCA
RC3H2 #3	CAGCAGTTGTCTGCCAATCTA
RC3H2 #4	AAGGAGGAATTTCGGAGTTAT
ZBTB41 #1	TAGGCGATGGCAGGTCATGAA
ZBTB41 #2	ATCCGTCATGATCACCTTACA
ZBTB41 #3	AAGCCTAATGTCTGTAATTAA
ZBTB41 #4	AGCGCTATTCAACAAAGTCTA
RNF19A #1	CAGGCTTTAGTGAATAACTTT
RNF19A #2	AAGATTCACAATCGCTATGAA
RNF19A #3	AACCTCAATGATAGCAGTTAA
RNF19A #4	ATCGGTGTTCCTATTATGTTA
CUL7 #1	CTGCTCTGCCATGGTAAGCAA
CUL7 #2	CCGGTCCATCTTTCAGCCCTA
CUL7 #3	CAGCTCGTCTACTTCACGAAA
CUL7 #4	CTGCAGGTTCTCAGTAGTCGA
RNF165 #1	CAGCACTATCAGCATTACCTA
RNF165 #2	CCCACAAGTATAAGAAGCGAA
RNF165 #3	CCGAGGACGCCAATCAATCAA
RNF165 #4	ATGGTCGTCCATGAAATCCGA

Supplementary Table 3. (continued)

**siRNA**	**Target sequence**
ZBTB40 #1	CTCCTACGACTCGGCCTATAA
ZBTB40 #2	CCTGTTGAGGCTGTACCAATA
ZBTB40 #3	CCGGCGCTGTTGAATGGCGAA
ZBTB40 #4	ATGATGTACACGGGCAAACTA
NUP43 #1	GTGCAATAAAGTAGAATTCTA
NUP43 #2	AAGACCGAATTGAAATCACAA
NUP43 #3	AGCAAACTATTTCCAAACTAA
NUP43 #4	CAGGGATAATAATGAACTAAT
ZBTB44_#1	CACCCGTATTCAGAACCTAAA
ZBTB44_#2	ACCGGACTACTTAAACCAGGA
ZBTB44_#3	CTGGCCCATCTGAGACCGGTA
ZBTB44_#4	CACAGCCACTCTATCAATTAA
RABGEF #1	AAGCCTCCGAATCAACCGTAA
RABGEF #2	CAGAAGGTGATAAGTTACTAT
RABGEF #3	AACCGGCAAACCAGCATTGAA
RABGEF #4	AACCTCGGGACTATTCATATT

Supplementary Table 3| List of siRNAs. The list shows target sequences of siRNA.

Supplementary References

1 Inoko, A. *et al.* Trichoplein and Aurora A block aberrant primary cilia assembly in proliferating cells. *J. Cell Biol.* **197**, 391-405 (2012).

2 Nishizawa, M. *et al.* Identification of trichoplein, a novel keratin filament-binding protein. *J. Cell Sci.* **118**, 1081-1090 (2005).

3 Ibi, M. *et al.* Trichoplein controls microtubule anchoring at the centrosome by binding to Odf2 and ninein. *J. Cell Sci.* **124**, 857-864 (2011).

4 Sorokin, S. P. Reconstructions of centriole formation and ciliogenesis in mammalian lungs. *J. Cell Sci.* **3**, 207-230 (1968).

5 Sorokin, S. Centrioles and the formation of rudimentary cilia by fibroblasts and smooth muscle cells. *J. Cell Biol.* **15**, 363-377 (1962).

6 Maruyama, Y. *et al.* Human Gene and Protein Database (HGPD): a novel database presenting a large quantity of experiment-based results in human proteomics. *Nucleic Acids Res.* **37**, D762-766 (2009).

7 Li, W. *et al.* Genome-wide and functional annotation of human E3 ubiquitin ligases identifies MULAN, a mitochondrial E3 that regulates the organelle's dynamics and signaling. *PLoS One* **3**, e1487 (2008).

8 Bosu, D. R. & Kipreos, E. T. Cullin-RING ubiquitin ligases: global regulation and activation cycles. *Cell Div.* **3**, 7 (2008).

9 Peters, J. M. The anaphase promoting complex/cyclosome: a machine designed to destroy. *Nat. Rev. Mol. Cell. Biol.* **7**, 644-656 (2006).
